# Modeling, virtual screening, and enzymatic docking of trehalose 6‐phosphate phosphatase and evaluation of the insecticidal effect of phthalimide, *N*‐(*p*‐tolylsulfonyl) on *Aedes aegypti* (Diptera: Culicidae)

**DOI:** 10.1002/ps.8841

**Published:** 2025-04-23

**Authors:** Raquel Jemima Viana Lima, Fernando Berton Zanchi, Railton Marques de Souza Guimarães, Alexandre de Almeida e Silva

**Affiliations:** ^1^ Departamento de Biologia Programa de Pós‐Graduação em Conservação e uso de Recursos Naturais, Universidade Federal de Rondônia Porto Velho Brazil; ^2^ Laboratório de Bioinformática e Química Medicinal Fundação Oswaldo Cruz Porto Velho Brazil; ^3^ Programa de Pós‐Graduação em Biologia Experimental Porto Velho Brazil; ^4^ Laboratório de Bioecologia de Insetos Universidade Federal de Rondônia Porto Velho Brazil

**Keywords:** larvicide, metabolism, trehalose, *in silico*

## Abstract

**BACKGROUND:**

*Aedes aegypti* Linnaeus is a medically important vector because of its role in transmitting several arboviruses. Trehalose‐6‐phosphate phosphatase (TPP), an enzyme from the trehalose pathway, was the focus of this study, which aimed to model it, perform molecular docking and select potential ligands to evaluate their larvicidal and adulticidal activity on the mosquito.

**RESULTS:**

Because no TPP structure for *A. aegypti* was described, the modeling was done by homology, using the TPP from *Mycobacterium tuberculosis* Zopf as a template, with 31% similarity. Following virtual screening, a search for TPP‐like molecules on PubChem resulted in 227 molecules, and phthalimide, *N*‐(*p*‐tolylsulfonyl) (PNT) was selected and tested *in vivo*. Larvicidal tests were conducted in 24‐well plates, and adulticidal tests used sugar baits with several concentrations (5 to 100 ppm) of PNT. In larval tests, mortality ranged from 38% to 52% at 24 h and reached 92% at 100 ppm PNT after 72 h. Larval mortality progressively increased over 96 h, with estimated lethal concentration (LC_50_ and LC_90_) values after 48 h of 18 and 198 ppm respectively. In adulticidal tests, despite high bait uptake, acute ingestion of PNT did not cause mortality in adult mosquitoes.

**CONCLUSION:**

PNT demonstrated larvicidal activity against *A. aegypti*, suggesting that mosquito TPP could be a target in the search for new‐generation insecticides. © 2025 The Author(s). *Pest Management Science* published by John Wiley & Sons Ltd on behalf of Society of Chemical Industry.

## Graphical abstract

Schematic diagram of the experimental procedures used to perform the bioinformatic steps and insecticidal assays using trehalose 6‐phosphate phosphatase of *Aedes aegypti* and the molecule phthalimide, *N*‐(*p*‐tolylsulfonyl).

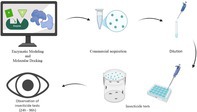

## 1 INTRODUCTION

Identifying molecules that target specific pathways in insects can help develop next‐generation insecticides with unique mechanisms of action. This approach can limit resistance to conventional insecticides and reduce impacts on non‐target organisms. The focus on metabolic pathway targets is promising because it deprives mosquitoes of important nutrients, altering their development.^1^

Among these targets, those linked to carbohydrate metabolism, such as trehalose, have been successfully explored.^2^ Trehalose is a disaccharide composed of two glucose molecules and is the primary sugar found in hemolymph. It has several functions in mosquitoes, such as providing energy for flight, metamorphosis, post‐stress recovery, and reproduction.^3^ Trehalose phosphate synthase (TPS) catalyzes the condensation of glucose‐6‐phosphate and uridine diphosphate‐glucose to generate trehalose‐6‐phosphate in the fat body. Trehalose‐6‐phosphate is then dephosphorylated by the protein phosphatase [trehalose‐6‐phosphate phosphatase (TPP)] encoded by the TPP gene to generate trehalose.^4^, ^5^ These two enzymes (TPS and TPP) work sequentially, so the fusion of the TPS/TPP gene may facilitate rapid trehalose synthesis to aid in survival under stress conditions.^5^

It has been reported that compounds with the substructure of phthalimide, a bicyclic non‐aromatic nitrogen heterocycle, have various biological activities and have been tested against viruses (human immunodeficiency virus), tumors, diabetes, multiple myeloma, convulsions, inflammation, pain and bacterial infections, among others.^6^ It has been found that TPP from the lepidopteran *Helicoverpa armigera* Hubner is inhibited by *N*‐(phenylthio)phthalimide (NPP), and the *in vivo* inhibition of trehalose synthesis altered metamorphosis, reduced larval weight and size, and decreased overall fitness in this insect, suggesting its potential use in insect controls.^3^ Therefore, this study aimed to model the trehalose‐6‐phosphate phosphatase of *A. aegypti* (AaeTPP), and perform virtual screening, molecular docking, and molecular dynamics (MD) simulation of potential NPP derivatives as ligands for AaeTPP, and evaluate the larvicidal and adulticidal effects of phthalimide, *N*‐(*p*‐tolylsulfonyl)‐ (PNT).

## 2 MATERIALS AND METHODS

### 2.1 Modeling of trehalose‐6‐phosphate phosphatase B from *Aedes aegypti*

The primary structure of TPP from *A. aegypti* (AaeTPP; GenBank code XP_001661021.2) is not deposited in the Protein Data Bank (PDB).^7^ Therefore, it was necessary to model it using a similarity‐based modeling approach utilizing the Blastp program^8^ to find possible templates and Modeller 10.3^9^ to model. A total of 1000 structures were generated and the structure with the lowest discrete optimized protein energy (DOPE) was selected as a valid and useful structure for molecular docking experiments. The modeled structure was validated using the programs Procheck^10^ and Verify3D,^11^, ^12^ along with calculating the root mean square deviation (RMSD) between the target structure and its template.

### 2.2 Virtual screening, docking and MD of AaeTPP

Molecules to be evaluated in virtual screening (*VS*) through docking were selected from the PubChem small molecule database.^13^ The structure of the substrate trehalose‐6‐phosphate (T6P; CID: 122336) was used as a lead in a search for similar compounds with a Tanimoto index of 95%.^14^ In addition, molecules with 90% similarity to NPP (CID: 5127161) were selected. This latter selection was inspired by the work of Cross *et al*.,^15^ which highlighted the inhibition of nematode TPP by NPP *in vitro*.

The molecules were prepared and docked using Autodock4.6^16^ with the PyRx interface.^17^ The three‐dimensional structures were minimized using the universal force field, followed by conversion to the PDBQT format. The docking simulation grid was established at the enzyme's active site, determined by comparison with the structure of TPP from *Salmonella typhimurium* Theobald Smith (PDB: 6UPC), which contains a crystallized trehalose‐6‐sulfate molecule (CID: 132502046). The Lamarckian genetic algorithm was employed for docking with 50 runs per ligand, an initial population of 200 randomly generated positions, an energy evaluation of 2.5 × 10^6^, 27 000 iterations with a mutation rate of 0.02, a crossover of 0.8, and an elitism value of 1. Molecules with the best interaction energies with AaeTPP, compared with its substrate (T6P), were selected for commercial acquisition. Interaction analyses and atomic proximity were performed using UCSF Chimera.^18^

The substrate, the NPP lead molecule, and the molecule with the best docking interaction energy, and which were commercially acquired, were selected for MD simulation in GROMACS 2024.2^19^ with the visual dynamics interface^20^ to generate scripts. The Amber99 force field^21^ was used. Partial charges and ligand topologies were obtained by Acpype^22^ using the ANTECHAMBER module.^23^ Electrostatic interactions were treated with the particle mesh Ewald algorithm using a cutoff of 12 Å. Each system was simulated under periodic boundary conditions in a cubic box with dimensions automatically defined, considering 2 Å from the outermost protein atoms in all Cartesian directions. The simulation box was filled with TIP3P water molecules.^24^ Subsequently, a two‐step energy minimization procedure was performed (2000 steps of steepest descent and 2000 steps of conjugate gradient, or until the system reached a force resistance below 1000 kJ/mol/nm). Next, initial atomic velocities were assigned using the Maxwell–Boltzmann distribution corresponding to a temperature of 300 K. All systems were equilibrated in two successive constant‐temperature, constant‐volume ensemble (NVT) and constant‐temperature, constant‐pressure ensemble (NPT) equilibration simulations, each lasting 200 ps. After this, all systems were simulated without restraints at 300 K in the Gibbs ensemble with a pressure of 1 atm, using isotropic coupling. All chemical bonds involving hydrogen atoms were restricted using the SHAKE algorithm,^25^ and the time step was set to 2 fs. Finally, three independent MD runs of 150 ns for each complex were conducted.

Simulation trajectories were analyzed using GROMACS package tools.^19^ RMSD and root mean square fluctuation (RMSF) were calculated separately for each system, fitting their heavy atoms and using the initial production dynamics structure as a reference. Hydrogen bonds were calculated intramolecularly for the protein and between the protein and ligand complexes. A hit was considered when the distance between two polar heavy atoms, with at least one hydrogen atom attached, was <3.5 Å and with an H donor angle >120°. The radius of gyration (Rg) was also calculated. Furthermore, interaction analysis of AaeTPP with most significant ligands was performed using Molecular Mechanics Poisson‐Boltzmann Surface Area (MMPBSA)^26^ for both the docked structure and the last 50 ns.

### 2.3 *Aedes aegypti* larvae and adults rearing for experiments

Mosquitoes from the Insect Bioecology Laboratory colony at the Federal University of Rondônia were used for the experiments, and rearing was performed as described by Barbosa *et al*.^27^ Briefly, the eggs were placed in 30 × 22 × 8 cm plastic trays containing 1 L of filtered water. After 24 h, the hatched larvae [first instar (L1)] were counted and transferred to 24‐well plates. Larvae not selected for larvicidal tests were fed crushed and diluted Tetramin® flakes until the second instar (L2). When the larvae reached the third instar (L3), they were transferred to 2‐L plastic cages (17 × 15 cm) with 1 L of filtered water and fed with Reptolife® granules. Once adults emerged, they were moved to screened cages (17 × 15 cm), and after 48 h, females were transferred to test cages.

### 2.4 Larvicidal tests

A PNT sample was obtained from Mcule (https://mcule.com), weighed and dissolved in dimethyl sulfoxide (DMSO) to prepare the stock solution. Three replicates were performed on 24‐well plates with three repetitions using different mosquito generations (*N* = 9), each with a final volume of 1 mL and containing five L1 larvae (Pridgeon *et al*. 2009)^28^ at concentrations of 100, 50, 25, and 10 ppm. The control contained only 1% DMSO. Crushed and powdered Tetramin® flakes were provided 24 h after the experiment began. Mortality was recorded daily over a period of 24 to 96 h.

### 2.5 Adulticidal feeding tests

Sugar baits (10% sucrose) containing different concentrations (100, 50, 25, and 10 ppm) of PNT were prepared. Approximately 500 μL of bait was applied as droplets on the mesh screen of 500‐mL plastic cages (8.5 cm × 9.5 cm) containing ten adult nulliparous *Ae. aegypti* females, aged 2–7 days and fasted for 48 h. The control used droplets containing only 10% sucrose. Mosquito engorgement was observed after 60 min and recorded. Mosquitoes were then fed with 10% sucrose soaked in cotton. The mortality of engorged females was monitored and recorded over a 96‐h period. The experiments were repeated three times using different mosquito generations, with three replicates per experiment.

### 2.6 Statistical analysis

The effects of PNT concentrations and time on *Ae. aegypti* larval and adult mortality were analyzed using two‐factor analysis of variance (ANOVA) with a mixed‐effects model and Geisser–Greenhouse correction, with comparisons made by Tukey's test at 5%. Bait engorgement was analyzed by one‐factor ANOVA. All tests were performed using Prism 10 (GraphPad). Lethal concentration calculations were performed by Probit analysis using a spreadsheet.^29^

## 3 RESULTS

### 3.1 Modeling of trehalose‐6‐phosphate phosphatase B of *Aedes aegypti*

**Figure 1:**
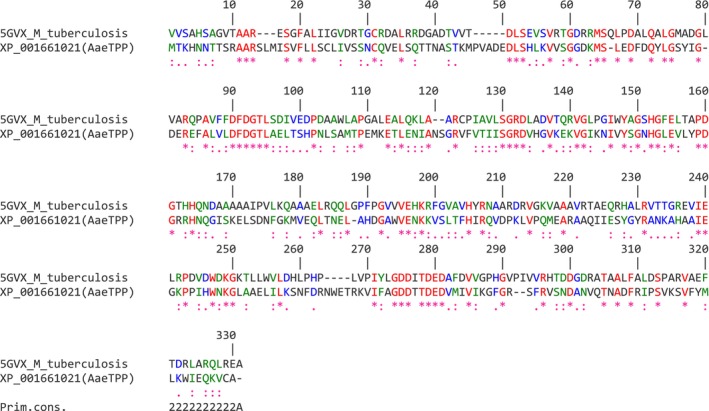
Alignment between part of the primary structure of the trehalose phosphate phosphatase template from *Mycobacterium tuberculosis* (PDB: 5GVX) and the target primary structure of trehalose‐6‐phosphate phosphatase of *Aedes aegypti* (AaeTPP). Dashes represent the gaps between the two structures. Asterisks indicate identities (amino acids in red), colons indicate strong similarity (amino acids in green), and periods (.) indicate weak similarity (amino acids in blue). The identity score is 30.8%.

The best template structure found for modeling was the TPP from *M. tuberculosis* (PDB: 5GVX), which has 30.8% similarity with AaeTPP (Fig. 1). The modeled structure was validated, and Procheck showed 91.8% of amino acids in favorable regions and none in disallowed regions (Supporting Information, Fig. S1(A)). The RMSD between the template and the modeled target structure was 0.603 Å, indicating excellent overlap between the enzymes (Supporting Information, Fig. S1(B)). The Verify3D assessment was also positive, showing 80.23% of amino acids with scores above 0.1 (Data not shown), further indicating good structure quality.

### 3.2 Virtual screening and molecular docking against AaeTPP

The search for T6P analogs in a two‐dimensional structure on PubChem using 95% similarity in the Tanimoto index resulted in 227 molecules, but only 166 had 3D structures (Supporting Information, Table S1). The same search with 90% similarity in the Tanimoto index for NPP analogs resulted in 163 molecules, with 151 having 3D structures (Supporting Information, Table S2). Therefore, 317 (166 + 151 molecules) molecules were tested against modeled AaeTPP. The simulation grid was positioned around the active site of modeled AaeTPP, aligned with the *Salmonella typhimurium* TPP structure (PDB: 6UPC), which has a crystallized trehalose‐6‐sulfate ligand. The grid center was −1.945 (X), −7.647 (Y) and −57.787 (Z), with dimensions of 51 Å (X), 65 Å (Y) and 51 Å (Z). A summary of the virtual screening results via docking can be found in Table 1, with rankings, energy, name, and chemical structure.

**Table 1:** List of some analogs that presented better binding energies than the crystallized ligand trehalose (CID: 122336) in the *VS* test

Ranking	Ligand (CID)	Name	Binding energy (kcal mol^−1^)	Structure
1	214 133 (PNT)	Phthalimide, *N*‐(*p*‐tolylsulfonyl)	−8.36	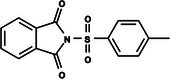
2	144 601 888	1‐Oxo‐2‐(2‐phenylacetyl)‐1,2‐benzothiazol‐3‐one	−8.04	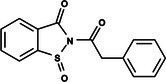
3	87 754 427	2‐(1‐Cyclohexylcyclohexyl)sulfanylisoindole‐1,3‐dione	−8.03	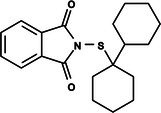
4	101 547 804	2‐(4‐Iodophenyl)sulfonylisoindole‐1,3‐dione	−7.91	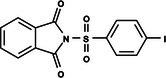
5	229 606	2‐(Benzenesulfonyl)isoindole‐1,3‐dione	−7.90	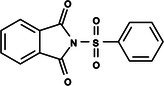
6	12 462 255	2‐Cyclohexylsulfinylisoindole‐1,3‐dione	−7.87	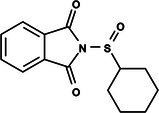
7	86 166 960	2‐(3‐Methylphenyl)sulfonylisoindole‐1,3‐dione	−7.86	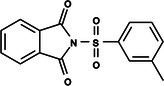
8	4 148 442	2‐(4‐Fluorophenyl)sulfonylisoindole‐1,3‐dione	−7.84	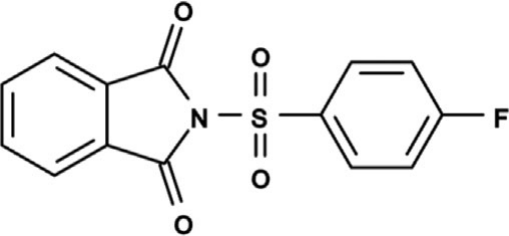
9	118 724 008	2‐[2‐(2‐methylphenyl)acetyl]‐1,2‐benzothiazol‐3‐one	−7.82	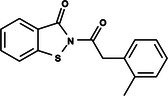
10	14 912 179	2‐[(*E*)‐1‐chloro‐1‐phenylprop‐1‐en‐2‐yl]sulfanylisoindole‐1,3‐dione	−7.79	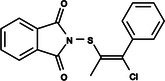
89	5 127 161 (NPP)	*N*‐(Phenylthio)phthalimide	−6.38	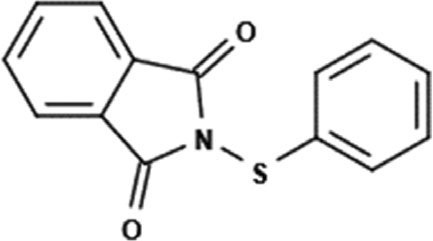
110	148 247 164	Bis[(3S,4S,5S,6R)‐3,4,5‐trihydroxy‐6‐(hydroxymethyl)oxan‐2‐yl] hydrogen phosphate	−6.18	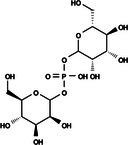
134	146 479 333	4‐[3,4,5‐Trihydroxy‐6‐(hydroxymethyl)oxan‐2‐yl]oxybutyl dihydrogen phosphate	−5.71	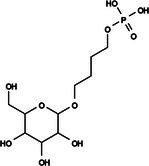
140	91 853 118	Man6P(a1‐2)a‐Man	−5.54	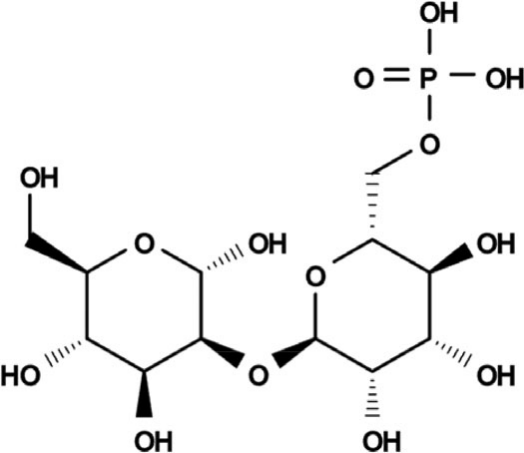
151	148 247 163	Bis[(3S,4S,5S,6R)‐3,4,5‐trihydroxy‐6‐(hydroxymethyl)oxan‐2‐yl] phosphate	−5.32	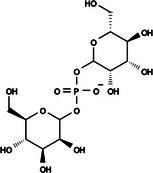
153	90 658 275	beta‐Maltose 6′‐phosphate	−5.20	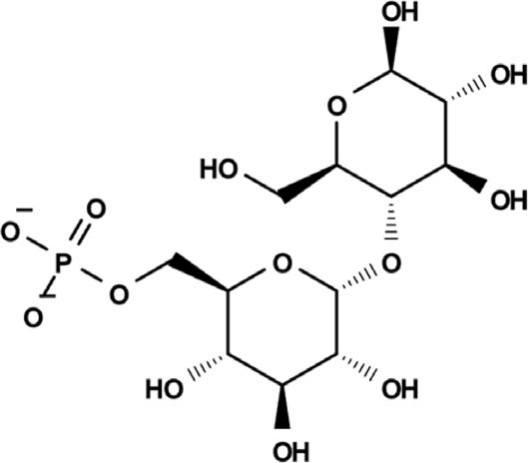
155	122 336 (T6P)	Trehalose 6‐phosphate	−5.18	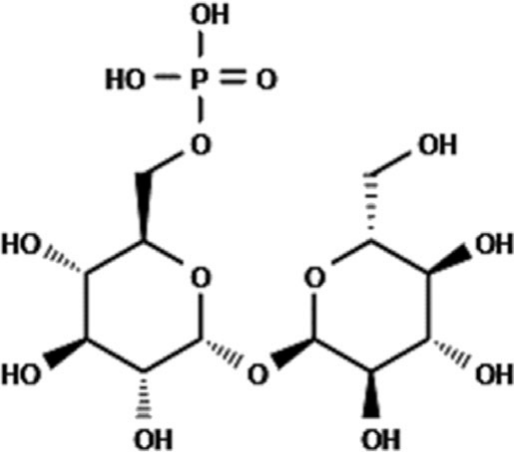

**Figure 2:**
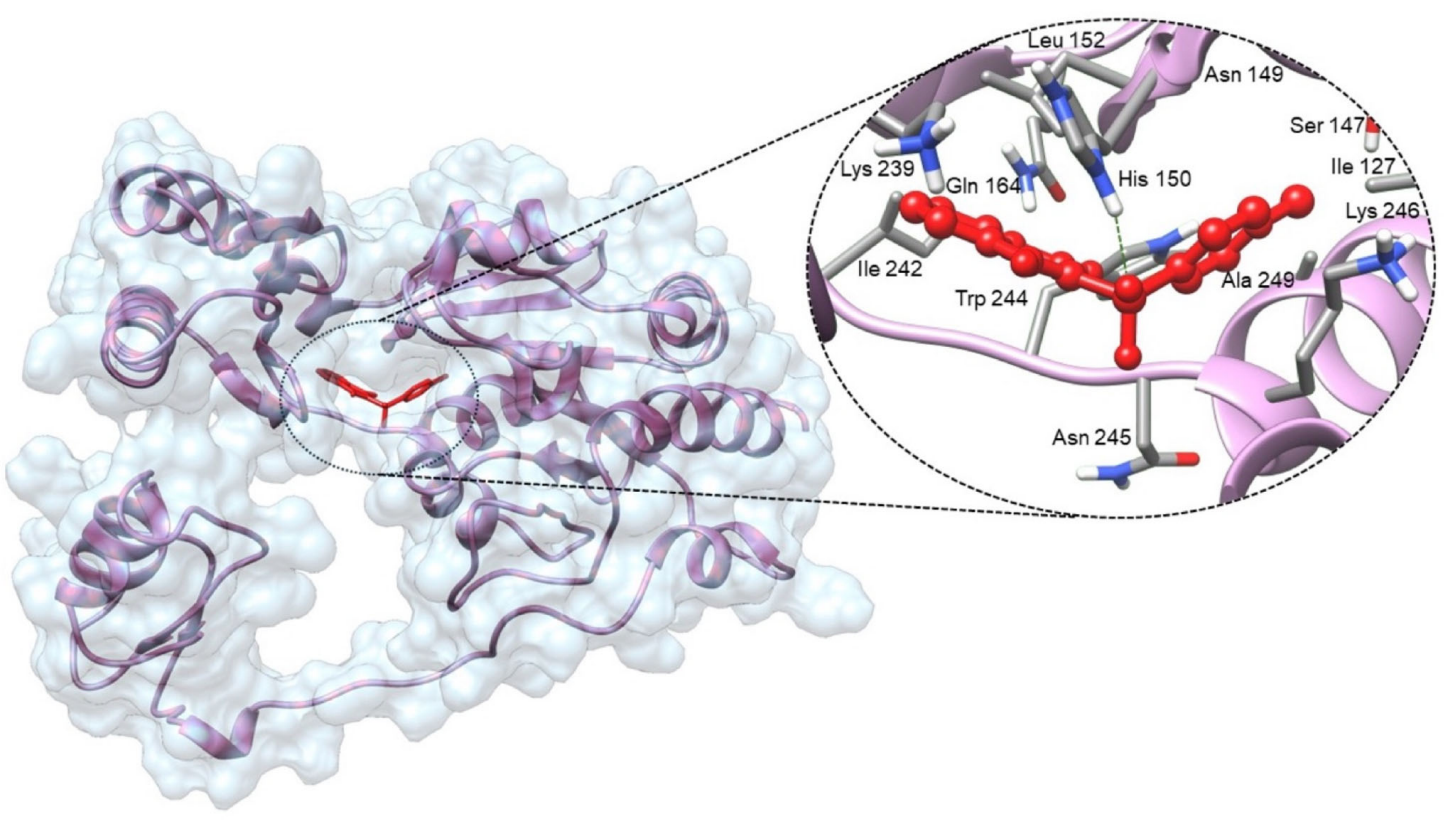
Interaction of phthalimide, *N*‐(*p*‐tolylsulfonyl) (CID: 214133) at the trehalose‐6‐phosphate phosphatase of *Aedes aegypti* binding site after docking. On the left, the surface range of the structure is shown with the ligand docked in its site. The binding energy calculated by the docking process is −8.36 kcal mol^−1^. On the right, without the surface, amino acids within 4 Å of the ligand are highlighted. A hydrogen bond between the ligand and histidine 150 is depicted by a green dashed line.

The T6P molecule, ranking 155 (CID: 122336) (Table 1), is the substrate of AaeTPP and served as a positive control. Its energy of −5.18 kcal mol^−1^ was used as a cutoff for selecting candidates during the virtual screening process. Of the 317 molecules tested, 154 exhibited interaction energies better than the control. Most of these molecules are NPP derivatives (CID: 5127161). Only the top ten in this group and the lead molecule NPP (rank 89) are shown in Table 1. The only T6P derivatives with compatible energies were the last four listed at the end of the table, with energies lower than the control substrate (rankings 151, 152, 153, 154), though they are unfavorable compared with the NPP derivatives. Only the first molecule, PNT (CID: 214133), was found commercially. Figure 2 shows PNT interacting with the active site amino acids of AaeTPP post docking.

### 3.3 Molecular dynamics

**Figure 3:**
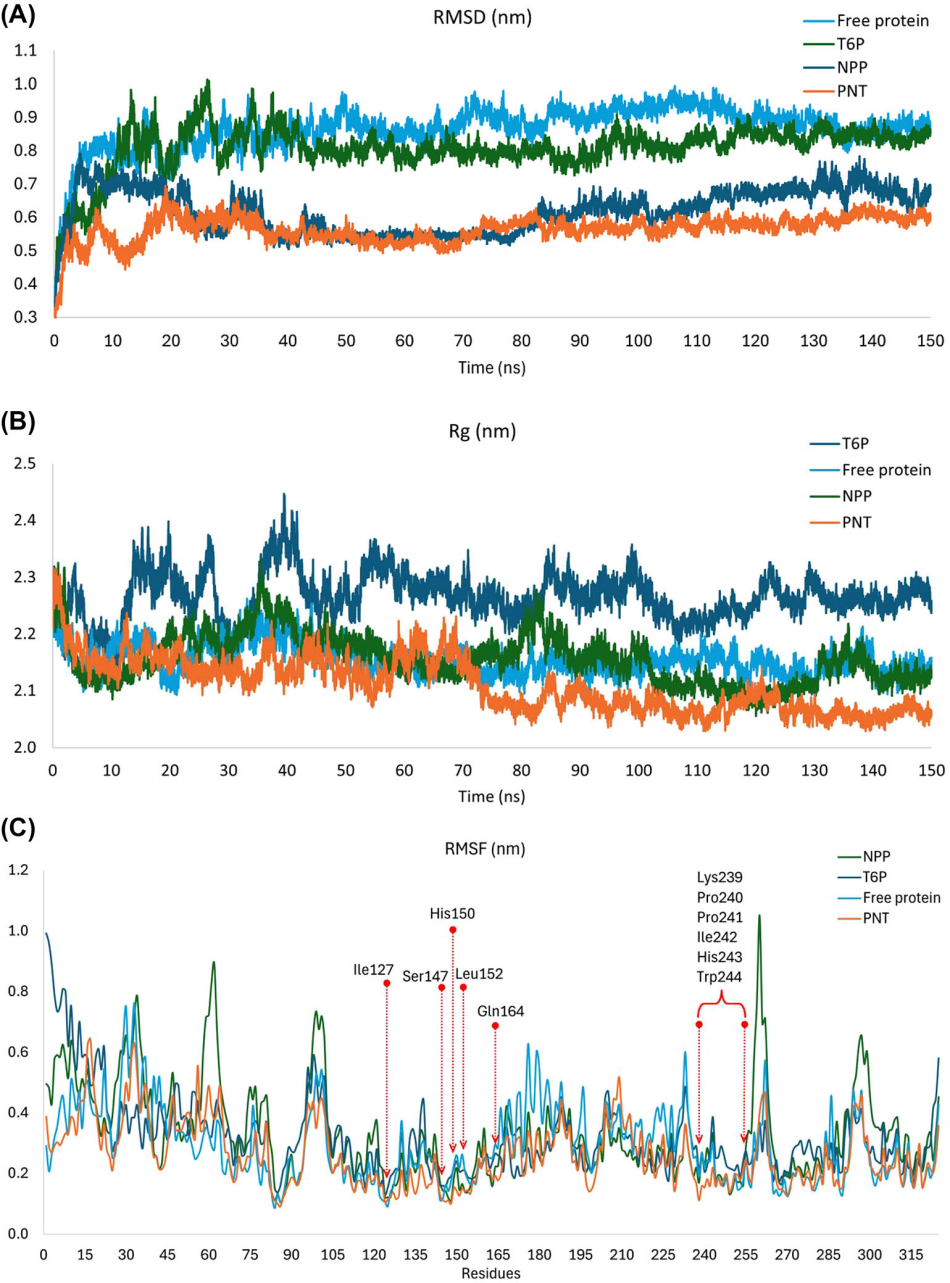
Analyses performed after the molecular dynamics simulation of all simulated systems. (A) Root mean square deviation (RMSD) showing how the phthalimide, *N*‐(*p*‐tolylsulfonyl) (PNT) molecule provides more stability to the protein than all other complexes and even the free protein. (B) Radius of gyration (Rg) showing how the PNT candidate provides the most compaction among all complexes or free protein. (C) Root mean square fluctuation (RMSF) showing how the PNT candidate provides less fluctuation of amino acids near the active site than other ligands or free protein.

Molecular dynamics simulation analyses allowed for the assessment of complex stability concerning the free protein, as well as between the control ligands and the drug candidate. Ultimately, the protein complexed with the PNT candidate showed greater stability than all other systems over the entire 150 ns simulation (Fig. 3(A)–(C)). Moreover, in Fig. 3(C), it is apparent that the active site amino acids were more stable in the presence of the PNT candidate than in any other system, whether it was the free protein, the T6P substrate, or the NPP lead compound.

Hydrogen bond analysis before MD simulation showed that T6P formed more bonds with AaeTPP than NPP and the PNT candidate (Supporting Information, Fig. S2). PNT maintained one bond almost permanently and, at times, up to three bonds. However, the T6P substrate can form up to nine bonds of this type (Supporting Information, Fig. S3). The energy profile calculated by the MMPBSA method showed that PNT had more favorable energy than the others (−16.68 kcal mol^−1^) (Supporting Information, Fig. S4).

### 3.4 Larvicidal activity of PNT on *Aedes aegypti*

**Figure 4:**
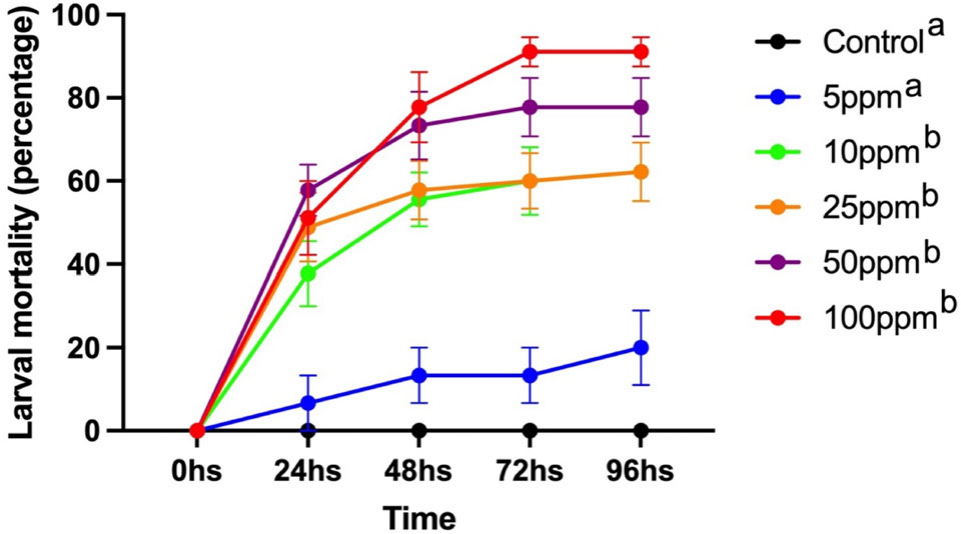
Larval mortality of *Aedes aegypti* larvae exposed to increasing concentrations of phthalimide, *N*‐(*p*‐tolylsulfonyl) from 24 to 96 h. Different letters indicate significant (*P* < 0.05) differences in larval mortality among concentration.

Increasing the concentration and exposure time of *A. aegypti* larvae to PNT resulted in a significant increase in larval mortality (*F* = 22.9, *P* < 0.0001 and *F* = 30.1, *P* < 0.0001, respectively). Concentrations between 10 and 100 ppm resulted in larval mortality ranging from 1.9 larvae (38%) to 2.9 larvae (58%) within 24 h of exposure, reaching 4.6 larvae (92%) at 100 ppm after 72 h. Overall, we observed a consistent increase in larval mortality from 24 to 96 h; namely, 2.0 (40%) to 3.3 larvae (66%) (Fig. 4).

The estimated lethal concentrations (LC) to kill 50% (LC_50_) tended to decrease over the observation period from 24 to 96 h, from 29 to 12 ppm. The LC values for killing 90% (LC_90_) of the larvae were 7 times higher than the LC_50_ at 24 h, reaching 11 times after 48 h (Table 2).

**Table 2:** Lethal concentration of phthalimide, *N*‐(*p*‐tolylsulfonyl) to first‐instar larvae of *Aedes aegypti* from 24 to 96 h

Time (h)	LC_50_ (LCI to UCI) ppm	LC_90_ (LCI to UCI) ppm
24	29 (15–53)	206 (110–385)
48	18 (9–35)	198 (101–386))
72	14 (8–25)	92 (53–161)
96	12 (7–23)	97 (53–179)

LC, lethal concentration; LCI, lower confidence interval; UCI, upper confidence interval.

**Figure 5:**
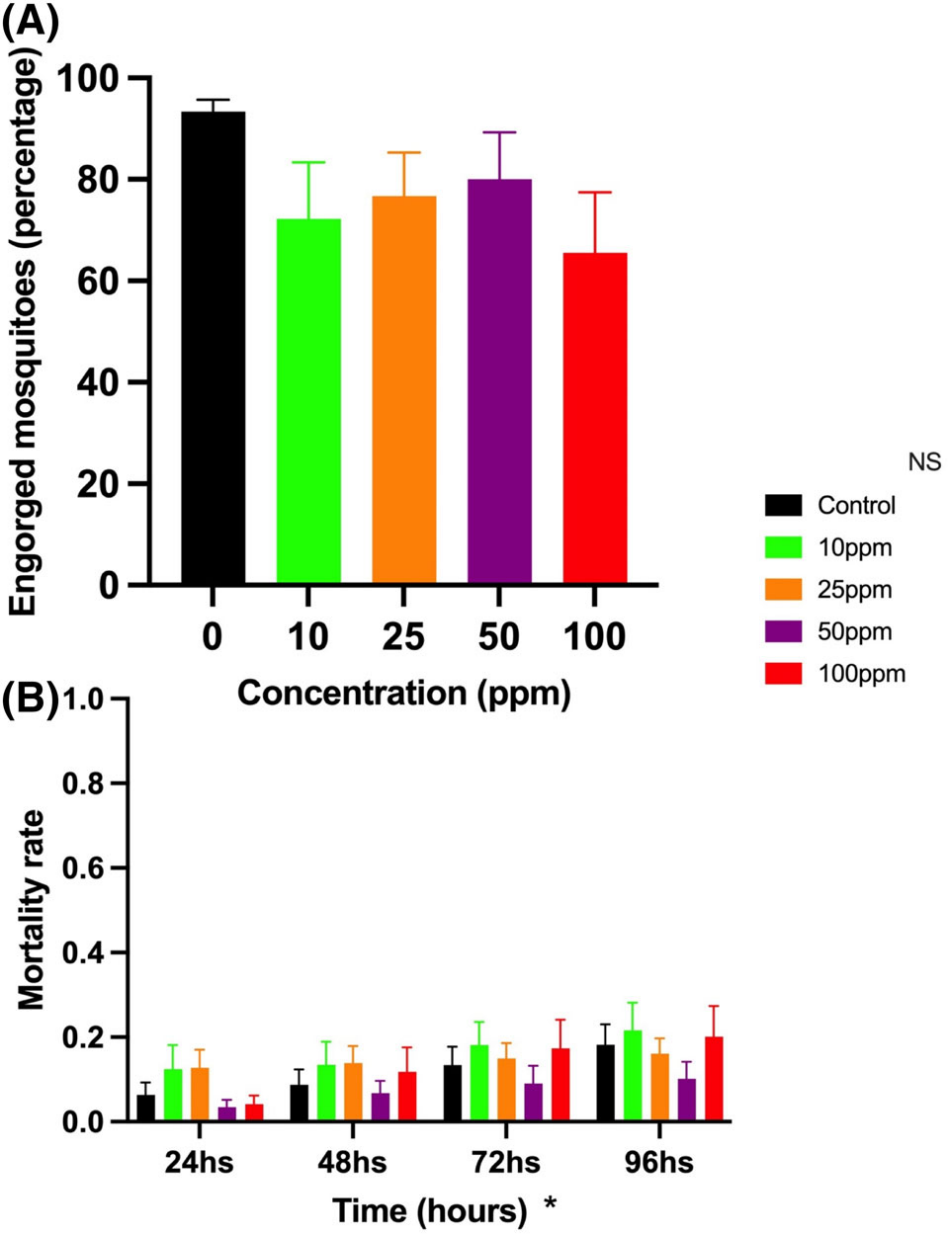
Engorgement (A) and mortality rate (B) of *Aedes aegypti* female adults fed with sugar baits containing increasing concentrations of phthalimide, *N*‐(*p*‐tolylsulfonyl) during adulticidal experiments from 24 to 96 h. NS, non‐significant (*P* > 0.05). *Significant differences in the mortality rate over time (*P* < 0.05).

In general, the PNT‐containing baits were engorged, and no significant differences were observed between the tested concentrations and the control (*F* = 1.24; *P* = 0.31). The number of engorged mosquitoes ranged from 6.5 (100 ppm) to 8.0 (50 ppm) (Fig. 5(A)). Although, in general, we observed a slight increase in adult mortality over the observation period (*F* = 14.8; *P* < 0.0001), from 0.074 (24 h) to 0.17 (96 h), there were no differences in the mortality of adults that engorged PNT‐containing baits and the control (*F* = 0.69; *P* = 0.60) (Fig. 5(B)).

## 4 DISCUSSION

This study illustrates the use of a rational protocol employing structural bioinformatics to discover new compounds, enabling the exclusion of unlikely candidates and directing resources toward the most promising options.^30^, ^31^, ^32^ At the end of the MD process, the PNT molecule (CID 214133) ranked highest with an energy of −8.36 kcal mol^−1^, surpassing even the lead molecule, NPP, with an energy of −6.37 kcal mol^−1^ (rank 89), and other analogs of the T6P substrate with an energy of −5.17 kcal mol^−1^ (rank 155). In the docking experiment, all molecules in the phthalimides family proved to be better ligands than the T6P substrate derivatives. All classical stability analyses of the protein performed after MD simulation (RMSD, Rg and RMSF) indicate that the PNT candidate confers greater stability to the protein than the T6P substrate used as a control. It also outperformed the lead molecule (NPP) used in the phthalimides selection filter.

When analyzing hydrogen bonds alongside the results of the MM‐PBSA method, we can conclude that the phthalimide derivatives primarily interact with AaeTPP through Van der Waals contributions, whereas T6P sugar derivatives rely on hydrogen bonds for their interaction.

For insecticidal activity, PNT induced larval mortality in *A. aegypti* at concentrations above 10 ppm, with efficacy increasing over time. The LC_50_ values decreased from 29 ppm at 24 h to 12 ppm at 96 h. Although no data are available for other mosquitoes or insects with aquatic stages, our results suggest that mosquito larvae are much more sensitive to the effects of this phthalimide derivative compared to other insects, such as the lepidopteran larvae *H. armigera*, *Mythimna separata* Walker, and *Spodoptera frugiperda* Smith, which were fed artificial diets containing increasing NPP concentrations (250–1000 ppm), about ten times higher than those used in this study.^5^

It has been reported that *H. armigera* larvae exhibited a 7%–26% reduction in body weight, as well as delayed development and metamorphosis suppression when fed 1000 ppm NPP^5^. Moreover, the survival of *H. armigera* larvae after 10 days of treatment was reduced to 60% (40% mortality), whereas *M. separata* larvae reached 65% mortality at 1000 ppm, and *S. frugiperda* larvae had less than 30% mortality at this concentration, suggesting that the effect of NPP is species‐dependent and that PNT might have an even greater impact, given that approximately 92% of *Ae. aegypti* larvae died after 72 h (3 days) of exposure to a concentration ten times lower than that of NPP.

Interestingly, the use of AaeTPP inhibitors like PNT in this study suggests that inhibiting this enzyme has a much stronger larvicidal effect than trehalase inhibitors such as validamycin A for the same species, as Marten *et al*. reared *A. aegypti* larvae at concentrations ranging from 1000 to 5000 ppm and observed only 9% larval mortality.^2^ However, other effects of chronic exposure of larvae to these inhibitors should be investigated, as Marten *et al*. reported that *A. aegypti* adults had more than 60% reduced flight capacity and a skewed sex ratio toward males when exposed to increasing concentrations of validamycin A.

Although sugar baits containing PNT were readily ingested by *A. aegypti* females, they did not result in adult mortality, unlike the effects observed in larvae. Our study, like that of Tellis *et al*.,^5^ chronically exposed larvae to trehalose metabolism enzyme inhibitors during development, whereas the purpose of toxic sugar baits is to provide a lethal dose after ingestion rather than chronic exposure. In addition, the bait composition includes 10% sucrose, which may have served as an additional energy source, even with decreased trehalose resulting from potential AaeTPP inhibition by PNT ingestion, as suggested for locusts (*Locusta migratoria* Linnaeus) injected with trehazolin, a trehalase inhibitor.^33^

Other application methods for adults could be considered, such as topical application. For instance, 300 ppm of phthalimide heterodimers caused 63%–93% mortality in the aphid *Lipaphis erysimi* Kaltenbach and the mite *Tetranychus cinnabarinus* Boisduval, although the target in these experiments was acetylcholinesterase. In addition, of 13 phthalimide derivatives tested topically on the fruit fly *Anastrepha suspensa* Loew, 3 caused more than 50% mortality when applied at concentrations between 0.7 and 1.4 μg per fly.^34^ Injecting 50 μg of the trehalase inhibitor trehazolin into locusts (*L. migratoria*) resulted in 50% insect mortality after 24 h, likely because of severe hypoglycemia.^33^ Thus, tarsal contact may be considered a potential delivery method for these inhibitors to adult mosquitoes in future experiments.

Although the mechanisms behind the larvicidal effect were not investigated in this study, chronic ingestion of PNT by *Ae. aegypti* larvae may have resulted in the accumulation of T6P, leading to harmful alterations in larval metabolism because in eukaryotes like *S. cerevisiae* and *Aspergillus fumigatus*, T6P sequestration of phosphate is believed to reduce ATP levels, directly inhibit hexokinases, and regulate glycolysis.^35^ Intestinal obstruction was also a hypothesis for early larval mortality of *Caenorhabditis elegans* during genetic screening for intestinal defects caused by the loss of function in the gob‐1 (TPP).^36^

Successful modeling of AaeTPP facilitated efficient virtual screening, including docking followed by MD simulations. Virtual screening revealed that although certain T6P substrate derivatives can bind to the enzyme, NPP derivatives exhibited stronger interactions. MD simulations further confirmed that, among all the ligands evaluated in this study, PNT is a promising inhibitor of AaeTPP *in silico* and exhibited larvicidal activity against *Ae. aegypti*, suggesting that mosquito TPP could be a target for next‐generation insecticides. However, future studies should include *in vitro* enzymatic inhibition assays and *in vivo* experiments using other TPP‐targeting compounds as positive controls to confirm that the insecticidal activity results from TPP inhibition.

Although PNT lacks deterrent effects and is readily ingested in sugar baits, it did not cause adult mortality, indicating that other delivery methods, such as tarsal contact, should be explored. Finally, given the sublethal effects observed in other studies on enzyme metabolism inhibitors in insects, including mosquitoes, assessing the biology and reproduction of adults derived from larvae chronically exposed to AaeTPP inhibitors may provide new insights for using this strategy for mosquito vector control.

## Supporting information


**Figure S1.** (A) Evaluation of the AaeTPP structure modeled in Procheck. Over 90% of the amino acids are in favorable regions, with none in forbidden regions. (B) Superposition of the template structure (PDB: 5GVX) in orange and the modeled AaeTPP structure in blue. The active site is indicated by dashed lines.
**Figure S2.** Interactions of T6P, NPP and PNT ligands with AaeTPP after the molecular docking process.
**Figure S3.** Analyses of the number of hydrogen bonds of the three ligands with AaeTPP during the 150 ns simulation.
**Figure S4.** Analyses of the binding free energy (ΔG) of the PNT candidate and the controls with AaeTPP.

**Table S1.** Trehalose‐6‐phosphate (T6P) analogs.
**Table S2.** N‐(phenylthio) phthalimide (NPP) analogs.

## Data Availability

The data that support the findings of this study are available from the corresponding author upon reasonable request.
