# Supplementary material for: Modeling, virtual screening, and enzymatic docking of trehalose 6‐phosphate phosphatase and evaluation of the insecticidal effect of phthalimide, N‐(p‐tolylsulfonyl) on Aedes aegypti (Diptera: Culicidae)
Source: Pest Manag Sci. 2025 Apr 23;81(8):4777–87. doi: 10.1002/ps.8841 (PMC12268809; doi:10.1002/ps.8841)

Supplementary Material

**Supp. Fig S1.** (A) Evaluation of the AaeTPP structure modeled in Procheck. Over 90% of the amino acids are in favorable regions, with none in forbidden regions. (B) Superposition of the template structure (PDB: 5GVX) in orange and the modeled AaeTPP structure in blue. The active site is indicated by dashed lines.

(A) (B)


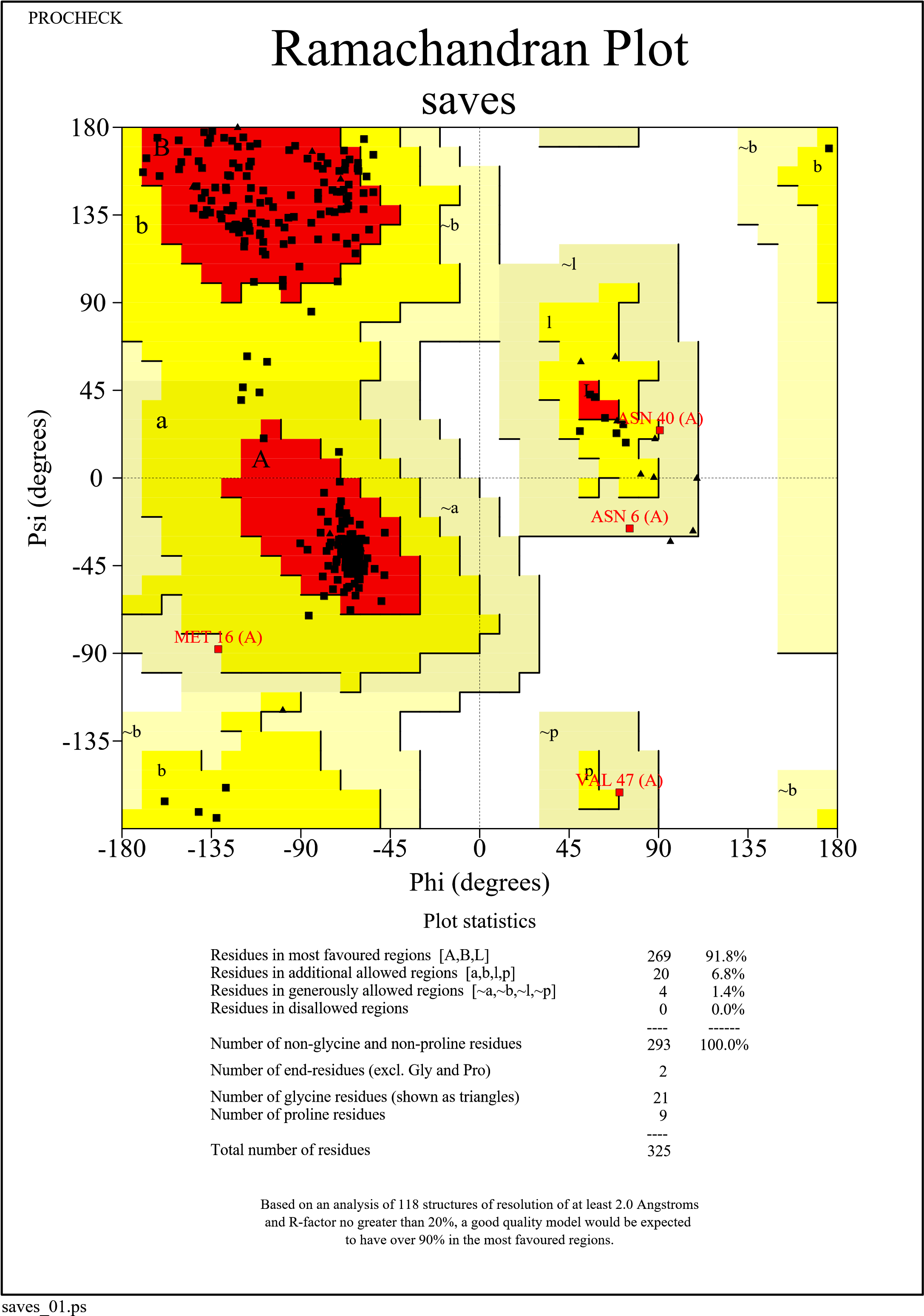


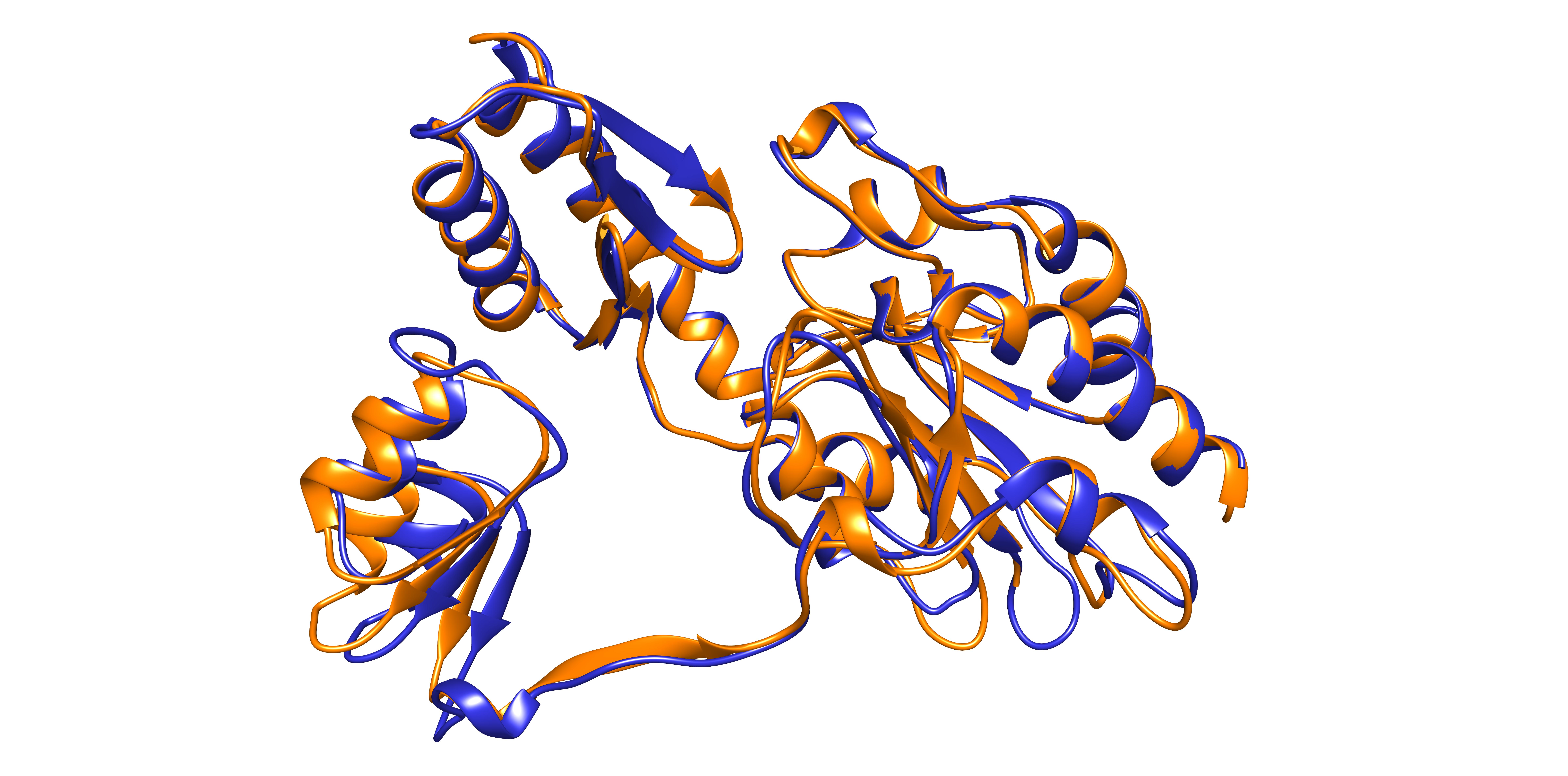


Active site

**Supp. Fig S2**. Interactions of T6P, NPP and PNT ligands with AaeTPP after the molecular docking process.


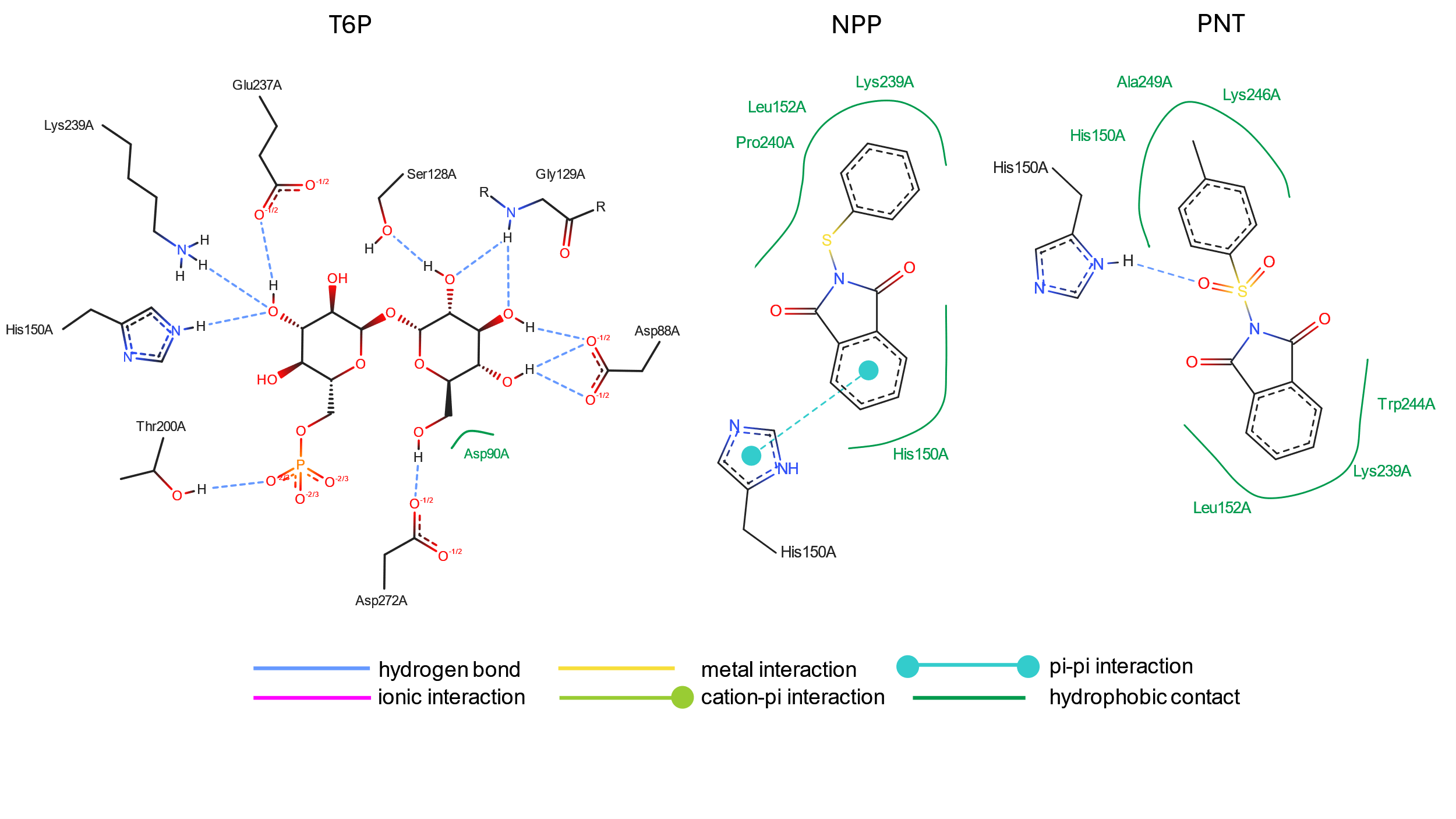


**Supp. Fig S3**. Analyses of the number of hydrogen bonds of the three ligands with AaeTPP during the 150 ns simulation.


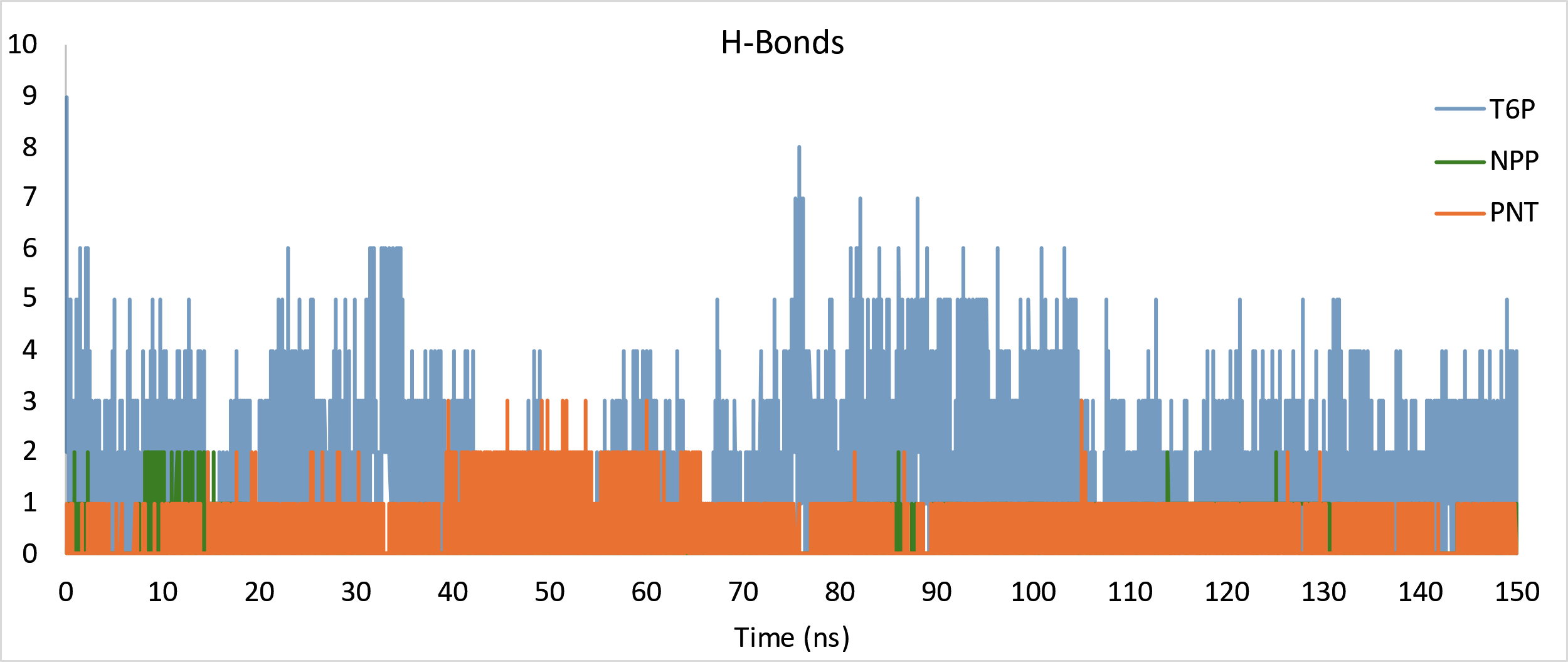


**Supp. Fig S4**. Analyses of the binding free energy (ΔG) of the PNT candidate and the controls with AaeTPP.


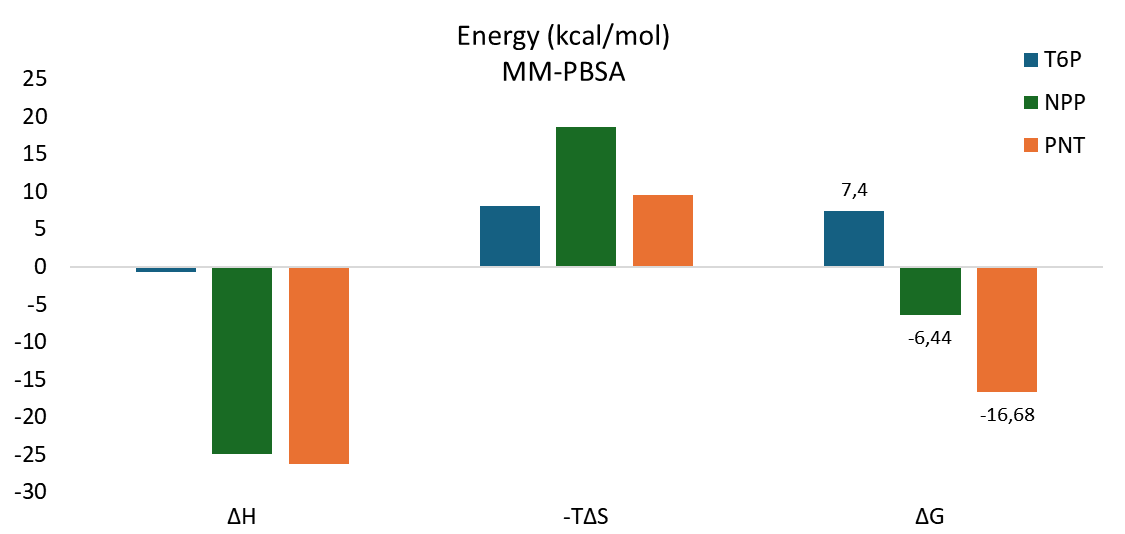

Supplement: Supplementary file 1 — Figure S1. (A) Evaluation of the AaeTPP structure modeled in Procheck. Over 90% of the amino acids are in favorable regions, with none in forbidden regions. (B) Superposition of the template structure (PDB: 5GVX) in orange and the modeled AaeTPP structure in blue. The active site is indicated by dashed lines. Figure S2. Interactions of T6P, NPP and PNT ligands with AaeTPP after the molecular docking process. Figure S3. Analyses of the number of hydrogen bonds of the three ligands with AaeTPP during the 150 ns simulation. Figure S4. Analyses of the binding free energy (ΔG) of the PNT candidate and the controls with AaeTPP. [file PS-81-4777-s001.docx]
