# Supplementary material for: Modeling, virtual screening, and enzymatic docking of trehalose 6‐phosphate phosphatase and evaluation of the insecticidal effect of phthalimide, N‐(p‐tolylsulfonyl) on Aedes aegypti (Diptera: Culicidae)
Source: Pest Manag Sci. 2025 Apr 23;81(8):4777–87. doi: 10.1002/ps.8841 (PMC12268809; doi:10.1002/ps.8841)
Supplement: Supplementary file 2 — Table S1. Trehalose‐6‐phosphate (T6P) analogs. Table S2. N‐(phenylthio) phthalimide (NPP) analogs. [file PS-81-4777-s002.docx]

**Supp. Table S1**. Trehalose-6-phosphate (T6P) analogs.

| **N** | **CID PubChem** | **Smiles** |
| --- | --- | --- |
| 1 | 440654 | O=P(O)(O)OC[C@H]1O[C@@H](O[C@H]2[C@H](O)[C@@H](O)[C@@H](O)O[C@@H]2CO)[C@H](O)[C@@H](O)[C@H]1O |
| 2 | 122336 | O=P(O)(O)OC[C@H]1O[C@H](O[C@H]2O[C@H](CO)[C@@H](O)[C@H](O)[C@H]2O)[C@H](O)[C@@H](O)[C@@H]1O |
| 3 | 25246105 | O=P([O-])([O-])OC[C@H]1O[C@H](O[C@H]2O[C@H](CO)[C@@H](O)[C@H](O)[C@H]2O)[C@H](O)[C@@H](O)[C@@H]1O |
| 4 | 440376 | O=P(O)(O)OC[C@H]1O[C@@H](O[C@H]2[C@H](O)[C@@H](O)[C@H](O)O[C@@H]2CO)[C@H](O)[C@@H](O)[C@@H]1O |
| 5 | 167489 | O=P(O)(O)O[C@H]1O[C@H](CO)[C@@H](O[C@H]2O[C@H](CO)[C@@H](O)[C@H](O)[C@H]2O)[C@H](O)[C@H]1O |
| 6 | 90658883 | O=P([O-])([O-])OC[C@@H](CO)O[C@H]1O[C@H](CO)[C@@H](O)[C@H](O)[C@H]1O |
| 7 | 86289748 | O=P([O-])([O-])OC[C@H]1O[C@@H](O[C@H]2[C@H](O)[C@@H](O)[C@@H](O)O[C@@H]2CO)[C@H](O)[C@@H](O)[C@H]1O |
| 8 | 56927942 | O=P([O-])([O-])O[C@H]1O[C@H](CO)[C@@H](O[C@H]2O[C@H](CO)[C@@H](O)[C@H](O)[C@H]2O)[C@H](O)[C@H]1O |
| 9 | 49852320 | O=P([O-])([O-])OC[C@H](O)CO[C@H]1O[C@H](CO)[C@H](O)[C@H](O)[C@H]1O |
| 10 | 49792005 | O=P([O-])([O-])OC[C@@H](CO)O[C@H]1O[C@H](CO)[C@H](O)[C@H](O)[C@H]1O |
| 11 | 44149386 | O=P(O)(O)O[C@@H]1O[C@H](CO)[C@@H](O[C@@H]2O[C@H](CO)[C@H](O)[C@H](O)[C@H]2O)[C@H](O)[C@H]1O |
| 12 | 440418 | O=P(O)(O)OC[C@@H](CO)O[C@H]1O[C@H](CO)[C@H](O)[C@H](O)[C@H]1O |
| 13 | 439874 | O=P(O)(O)OC[C@H]1O[C@H](O[C@H]2[C@H](O)[C@@H](O)[C@@H](O)O[C@@H]2CO)[C@H](O)[C@@H](O)[C@@H]1O |
| 14 | 164878 | O=P(O)(O)OC[C@H]1O[C@H](O[C@H]2O[C@H](COP(=O)(O)O)[C@@H](O)[C@H](O)[C@H]2O)[C@H](O)[C@@H](O)[C@@H]1O |
| 15 | 130561 | O=P(O)(O)OC[C@@H](O[C@@H]1O[C@H](CO)[C@@H](O)[C@H](O)[C@H]1O)[C@@H](O)[C@@H](O)CO |
| 16 | 129449 | O=P(O)(O)O[C@H]1[C@H](O)[C@@H](O)[C@H](O[C@@H](CO)[C@H](O)[C@H](O)CO)O[C@@H]1CO |
| 17 | 91860699 | O=P(O)(O)O[C@H]1[C@@H](O)[C@@H](CO)O[C@@H](O[C@H]2[C@H](O)[C@@H](O)[C@@H](O)O[C@@H]2CO)[C@@H]1O |
| 18 | 91858223 | O=P(O)(O)OC[C@H]1O[C@H](O)[C@H](O)[C@@H](O)[C@@H]1O[C@@H]1O[C@H](CO)[C@H](O)[C@H](O)[C@H]1O |
| 19 | 91851561 | O=P(O)(O)OC[C@H]1O[C@H](O[C@H]2[C@H](O)[C@@H](O)[C@@H](O)O[C@@H]2CO)[C@H](O)[C@@H](O)[C@@H]1O |
| 20 | 49852297 | O=P([O-])([O-])OC[C@H]1O[C@@H](O[C@H]2[C@H](O)[C@@H](O)[C@H](O)O[C@@H]2CO)[C@H](O)[C@@H](O)[C@@H]1O |
| 21 | 46224574 | O=P(O)(O)OC[C@H]1O[C@@H](O[C@H]2[C@H](O)[C@H](O)[C@@H](O)O[C@@H]2CO)[C@H](O)[C@@H](O)[C@H]1O |
| 22 | 44224013 | O=P(O)(O)OC[C@H](O)CO[C@H]1O[C@H](CO)[C@H](O)[C@H](O)[C@H]1O |
| 23 | 25200370 | [O-][P+]([O-])([O-])OC[C@H]1O[C@H](O[C@H]2[C@H](O)[C@@H](O)[C@@H](O)O[C@@H]2CO)[C@H](O)[C@@H](O)[C@@H]1O |
| 24 | 16048616 | O=P(O)(O)OC[C@@H](CO)O[C@H]1O[C@H](CO)[C@H](O)[C@H](O)[C@H]1O |
| 25 | 11761630 | O=P(O)(O)O[C@@H]1[C@@H](O)[C@@H](O[C@H]2O[C@H](CO)[C@@H](O)[C@H](O)[C@H]2O)O[C@H](CO)[C@H]1O |
| 26 | 5459947 | O=P(O)(O)OC[C@H]1O[C@@H](O[C@H]2[C@H](O)[C@@H](O)[C@@H](O)O[C@@H]2CO)[C@H](O)[C@@H](O)[C@@H]1O |
| 27 | 444691 | O=P(O)(O)OC[C@H]1O[C@H](O[C@@H]2O[C@H](CO)[C@@H](O)[C@H](O)[C@H]2O)[C@H](O)[C@@H](O)[C@@H]1O |
| 28 | 195740 | CCCO[C@H]1O[C@H](CO)[C@H](O[C@@H]2O[C@H](CO)[C@H](O)[C@H](O)[C@H]2O)[C@@](O)(OP(=O)(O)O)C1(O)O |
| 29 | 118796908 | O=P([O-])([O-])OC[C@H]1O[C@H](O[C@H]2[C@H](O)[C@@H](O)[C@@H](O)O[C@@H]2CO)[C@H](O)[C@@H](O)[C@@H]1O |
| 30 | 92449963 | O=P(O)(O)O[C@H]1O[C@H](CO)[C@@H](O[C@@H]2O[C@H](CO)[C@H](O)[C@H](O)[C@H]2O)[C@H](O)[C@H]1O |
| 31 | 91860613 | O=P(O)(O)OC[C@H]1O[C@H](OC[C@H]2O[C@H](O)[C@@H](O)[C@@H](O)[C@@H]2O)[C@@H](O)[C@@H](O)[C@@H]1O |
| 32 | 91860530 | O=P(O)(O)OC[C@H]1O[C@H](O[C@@H]2[C@H](O)[C@@H](O)O[C@H](CO)[C@H]2O)[C@@H](O)[C@@H](O)[C@@H]1O |
| 33 | 91856810 | O=P(O)(O)OC[C@H]1O[C@H](O)[C@@H](O[C@H]2O[C@H](CO)[C@@H](O)[C@H](O)[C@@H]2O)[C@@H](O)[C@@H]1O |
| 34 | 91853118 | O=P(O)(O)OC[C@H]1O[C@H](O[C@H]2[C@@H](O)[C@H](O)[C@@H](CO)O[C@@H]2O)[C@@H](O)[C@@H](O)[C@@H]1O |
| 35 | 91852343 | O=P(O)(O)OC[C@H]1O[C@H](O[C@H]2[C@@H](O)[C@H](O)[C@@H](CO)O[C@@H]2O)[C@@H](O)[C@@H](O)[C@@H]1O |
| 36 | 91847364 | O=P(O)(O)OC[C@H]1O[C@H](OC[C@H]2O[C@H](O)[C@@H](O)[C@@H](O)[C@@H]2O)[C@@H](O)[C@@H](O)[C@@H]1O |
| 37 | 91846983 | O=P(O)(O)OC[C@H]1O[C@H](O)[C@@H](O[C@H]2O[C@H](CO)[C@@H](O)[C@H](O)[C@@H]2O)[C@@H](O)[C@@H]1O |
| 38 | 91845785 | O=P(O)(O)OC[C@H]1O[C@H](O[C@@H]2[C@H](O)[C@@H](O)O[C@H](CO)[C@H]2O)[C@@H](O)[C@@H](O)[C@@H]1O |
| 39 | 90658884 | O=P(O)(O)OC[C@@H](CO)O[C@H]1O[C@H](CO)[C@@H](O)[C@H](O)[C@H]1O |
| 40 | 86289958 | O=P(O)(O[C@@H]1[C@@H](O)[C@H](O)[C@@H](CO)O[C@@H]1O)O[C@@H]1[C@@H](O)[C@H](O)[C@@H](CO)O[C@@H]1O |
| 41 | 60196241 | O=P(O)(O)OC[C@H]1O[C@@H](OC[C@H]2O[C@@H](O)[C@H](O)[C@@H](O)[C@@H]2O)[C@H](O)[C@@H](O)[C@@H]1O |
| 42 | 53914675 | O=P(O)(O)O[C@@H]1[C@@H](O)[C@@H](O[C@H]2[C@H](O)[C@@H](O)[C@H](O)O[C@@H]2CO)O[C@H](CO)[C@H]1O |
| 43 | 49873684 | O=P(O)(O)OC[C@H]1O[C@H](O[C@H]2O[C@H](CO)[C@@H](O)[C@H](O)[C@H]2O)[C@H](F)[C@@H](O)[C@@H]1O |
| 44 | 46936379 | O=P(O)(O)OC[C@@H]1O[C@H](O[C@H]2O[C@@H](CO)[C@H](O)[C@@H](O)[C@@H]2O)[C@@H](O)[C@H](O)[C@H]1O |
| 45 | 46931110 | O=P([O-])([O-])OC[C@@H](CO)O[C@@H]1O[C@H](CO)[C@@H](O)[C@H](O)[C@H]1O |
| 46 | 25244526 | O=P([O-])([O-])OC[C@H]1O[C@@H](O[C@H]2[C@H](O)[C@@H](O)[C@@H](O)O[C@@H]2CO)[C@H](O)[C@@H](O)[C@H]1O |
| 47 | 22842572 | O=P(O)(O)OC[C@H]1O[C@@H](O[C@H]2[C@H](O)[C@@H](O)[C@@H](O)O[C@@H]2CO)[C@H](O)[C@@H](O)[C@H]1O |
| 48 | 15942881 | O=P(O)(O)OC[C@@H](CO)O[C@@H]1O[C@H](CO)[C@@H](O)[C@H](O)[C@H]1O |
| 49 | 11037237 | O=P(O)(O)O[C@H]1[C@@H](O[C@H]2O[C@H](CO)[C@@H](O)[C@H](O)[C@H]2O)O[C@H](CO)[C@@H](O)[C@@H]1O |
| 50 | 153694965 | O=P([O-])([O-])O[C@H]1O[C@H](CO)[C@@H](O[C@@H]2O[C@H](CO)[C@H](O)[C@H](O)[C@H]2O)[C@H](O)[C@H]1O |
| 51 | 150869363 | O=P(O)(O)OC[C@@H](CO[C@H]1O[C@H](CO)[C@@H](O)[C@H](O)[C@H]1O)O[C@H]1O[C@H](CO)[C@@H](O)[C@H](O)[C@H]1O |
| 52 | 150680086 | O=P(O)(O)OC[C@H]1O[C@H](O)[C@H](OP(=O)(O)O)[C@@H](O)[C@@H]1O[C@@H]1O[C@H](CO)[C@H](O)[C@H](O)[C@H]1O |
| 53 | 148247163 | O=P([O-])(O[C@H]1O[C@H](CO)[C@@H](O)[C@H](O)[C@@H]1O)O[C@H]1O[C@H](CO)[C@@H](O)[C@H](O)[C@@H]1O |
| 54 | 146479333 | O=P(O)(O)OCCCCO[C@@H]1O[C@@H](CO)[C@H](O)[C@@H](O)[C@H]1O |
| 55 | 146479332 | O=P(O)(O)OCCO[C@H]1O[C@@H](CO)[C@@H](O)[C@H](O)[C@H]1O |
| 56 | 146037377 | O=P([O-])([O-])OC[C@H]1O[C@H](O[C@H]2O[C@H](CO)[C@@H](O)[C@H](O)[C@H]2O)[C@H](O)[C@@H](O)[C@@H]1O |
| 57 | 146017845 | O=P(O)(O)OC[C@H]1O[C@H](O[C@H]2O[C@H](CO)[C@@H](O)[C@H](O)[C@H]2O)[C@H](O)[C@@H](O)[C@@H]1O |
| 58 | 146017842 | O=P(O)(O)O[C@H]1[C@H](O)[C@@H](O)[C@@H](O[C@H]2O[C@H](CO)[C@@H](O)[C@H](O)[C@H]2O)O[C@@H]1CO |
| 59 | 146017837 | O=P(O)(O)O[C@H]1[C@@H](O[C@H]2O[C@H](CO)[C@@H](O)[C@H](O)[C@H]2O)O[C@H](CO)[C@@H](O)[C@@H]1O |
| 60 | 146017834 | O=P(O)(O)O[C@@H]1[C@@H](O)[C@@H](O[C@H]2O[C@H](CO)[C@@H](O)[C@H](O)[C@H]2O)O[C@H](CO)[C@H]1O |
| 61 | 141744233 | O=P(O)(O)O[C@]1(O)O[C@H](CO)[C@@H](O[C@@H]2O[C@H](O)[C@@H](O)[C@H](O)[C@H]2O)[C@@H](O)[C@H]1O |
| 62 | 141490213 | O=P(O)(O)OCCO[C@@H]1O[C@H](CO)[C@@H](O)[C@H](O)[C@H]1O |
| 63 | 141066703 | O=P(O)(O)O[C@]1(O[C@H]2[C@H](O)[C@@H](O)[C@H](O)O[C@@H]2CO)O[C@H](CO)[C@H](O)[C@H](O)[C@H]1O |
| 64 | 139935719 | O=P(O)(O)OC[C@H]1O[C@H](OP(=O)(O)O)[C@H](O)[C@@H](O)[C@@H]1O[C@@H]1O[C@H](CO)[C@H](O)[C@H](O)[C@H]1O |
| 65 | 129817303 | O=P(O)(O)O[C@@H]1[C@@H](O)[C@@H](O)O[C@H](CO)[C@H]1O[C@@H]1O[C@H](CO)[C@H](O)[C@H](O)[C@H]1O |
| 66 | 118050132 | O=P(O)(O)O[C@@H]1[C@@H](O)[C@@H](O[C@H]2O[C@H](CO)[C@@H](O)[C@H](O)[C@H]2O)O[C@H](CO)[C@H]1OP(=O)(O)O |
| 67 | 118050131 | O=P(O)(O)O[C@H]1[C@@H](O[C@H]2O[C@H](CO)[C@@H](O)[C@H](O)[C@H]2O)O[C@H](CO)[C@@H](O)[C@@H]1OP(=O)(O)O |
| 68 | 91862432 | O=P(O)(O)OC[C@H]1O[C@H](O[C@@H]2[C@@H](O)[C@H](O)[C@@H](CO)O[C@@H]2O)[C@H](O)[C@@H](O)[C@H]1O |
| 69 | 91862343 | O=P(O)(O)OC[C@H]1O[C@H](O[C@@H]2[C@@H](O)[C@H](O)[C@@H](CO)O[C@@H]2O)[C@H](O)[C@@H](O)[C@H]1O |
| 70 | 91862289 | O=P(O)(O)OC[C@H]1O[C@H](O[C@@H]2[C@@H](O)[C@H](O)[C@@H](CO)O[C@@H]2O)[C@H](O)[C@@H](O)[C@@H]1O |
| 71 | 91861404 | O=P(O)(O)OC[C@H]1O[C@H](O)[C@H](O[C@H]2O[C@H](CO)[C@@H](O)[C@H](O)[C@H]2O)[C@@H](O)[C@@H]1O |
| 72 | 91861085 | O=P(O)(O)OC[C@H]1O[C@@H](OC[C@H]2O[C@H](O)[C@H](O)[C@@H](O)[C@@H]2O)[C@H](O)[C@@H](O)[C@@H]1O |
| 73 | 91860050 | O=P(O)(O)O[C@@H]1[C@@H](O)[C@H](O[C@H]2[C@H](O)[C@@H](O)[C@@H](O)O[C@@H]2CO)O[C@H](CO)[C@@H]1OP(=O)(O)O |
| 74 | 91859710 | O=P(O)(O)OC[C@H]1O[C@@H](O[C@H]2[C@H](O)[C@H](O)[C@@H](O)O[C@@H]2CO)[C@H](O)[C@@H](O)[C@H]1O |
| 75 | 91856827 | O=P(O)(O)O[C@@H]1[C@H](O)[C@@H](O)[C@H](O[C@H]2[C@H](O)[C@@H](O)[C@@H](O)O[C@@H]2CO)O[C@@H]1CO |
| 76 | 91856511 | O=P(O)(O)OC[C@H]1O[C@H](OC[C@H](O)CO)[C@H](O)[C@@H](O)[C@@H]1O |
| 77 | 91855223 | O=P(O)(O)OC[C@H]1O[C@H](O)[C@H](O[C@H]2O[C@H](CO)[C@@H](O)[C@H](O)[C@H]2O)[C@@H](O)[C@@H]1O |
| 78 | 91854638 | O=P(O)(O)OC[C@H]1O[C@H](O[C@@H]2[C@@H](O)[C@H](O)[C@@H](COP(=O)(O)O)O[C@@H]2O)[C@H](O)[C@@H](O)[C@@H]1O |
| 79 | 91854231 | O=P(O)(O)OC[C@H]1O[C@H](O[C@H]2[C@@H](O)[C@H](O)[C@@H](COP(=O)(O)O)O[C@@H]2O)[C@@H](O)[C@@H](O)[C@@H]1O |
| 80 | 91854215 | O=P(O)(O)O[C@@H]1[C@@H](O)[C@H](O[C@@H]2O[C@H](CO)[C@H](O)[C@H](O)[C@H]2O)CO[C@H]1O |
| 81 | 91854193 | O=P(O)(O)OC[C@H]1O[C@@H](OC[C@H]2O[C@@H](O)[C@H](O)[C@@H](O)[C@H]2O)[C@H](O)[C@@H](O)[C@H]1O |
| 82 | 91853856 | O=P(O)(O)OC[C@H]1O[C@H](O[C@H]2[C@@H](OC[C@H](O)CO)O[C@H](CO)[C@@H](O)[C@@H]2O)[C@H](O)[C@@H](O)[C@H]1O |
| 83 | 91853415 | O=P(O)(O)OC[C@H]1O[C@H](O[C@@H]2[C@@H](O)[C@@H](O)O[C@H](CO)[C@H]2O)[C@H](O)[C@@H](O)[C@H]1O |
| 84 | 91853402 | O=P(O)(O)O[C@@H]1[C@@H](O)[C@H](O[C@H]2[C@H](O)[C@@H](O)[C@H](O)O[C@@H]2CO)O[C@H](CO)[C@@H]1OP(=O)(O)O |
| 85 | 91852845 | O=P(O)(O)OC[C@H]1O[C@@H](O)[C@H](O)[C@@H](O[C@@H]2O[C@H](CO)[C@H](O)[C@H](O)[C@H]2O)[C@H]1O |
| 86 | 91849634 | O=P(O)(O)OC[C@H]1O[C@H](O[C@@H]2[C@@H](O)[C@@H](O)O[C@H](CO)[C@H]2O)[C@H](O)[C@@H](O)[C@H]1O |
| 87 | 91848629 | O=P(O)(O)O[C@@H]1[C@@H](O)[C@@H](OC[C@H](O)CO)O[C@H](CO)[C@H]1O |
| 88 | 91847334 | O=P(O)(O)OC[C@H]1O[C@H](O[C@@H]2[C@@H](O)[C@H](O)[C@@H](COP(=O)(O)O)O[C@@H]2O)[C@H](O)[C@@H](O)[C@@H]1O |
| 89 | 91846266 | O=P(O)(O)OC[C@H]1O[C@H](O[C@@H]2[C@@H](O)[C@H](O)[C@@H](CO)O[C@@H]2O)[C@H](O)[C@@H](O)[C@@H]1O |
| 90 | 91845724 | O=P(O)(O)OC[C@H]1O[C@H](O[C@H]2[C@@H](O)[C@H](O)[C@@H](COP(=O)(O)O)O[C@@H]2O)[C@@H](O)[C@@H](O)[C@@H]1O |
| 91 | 91319661 | CO[C@H]1O[C@H](CO)[C@@H](O)[C@H](O[C@H]2O[C@H](COP(=O)(O)O)[C@@H](O)[C@H](O)[C@@H]2O)[C@@H]1O |
| 92 | 90806488 | O=P(O)(O)OC[C@H]1O[C@@H](O[C@H]2O[C@H](CO)[C@H](O)[C@H](O)[C@H]2O)[C@H](O)[C@@H](O)[C@H]1O |
| 93 | 90280322 | O=P(O)(O)O[C@@H]1O[C@H](CO)[C@@H](O[C@H]2O[C@H](CO)[C@@H](O)[C@H](O)[C@H]2O)[C@H](OP(=O)(O)O)[C@H]1OP(=O)(O)O |
| 94 | 87347304 | O=P(O)(O)O[14C@@H]1[C@@H](O)[14C@@H](O[14C@H]2O[C@H](CO)[C@@H](O)[C@H](O)[C@H]2O)O[14C@H]([14CH2]O)[14C@H]1O |
| 95 | 71281515 | O=POC[C@H]1O[C@@H](O[C@H]2O[C@H](CO)[C@@H](O)[C@H](O)[C@@H]2O)[C@@H](O)[C@@H](O)[C@@H]1O |
| 96 | 70912446 | O=P(O)(O)OC[C@@H]1O[C@H](O[C@H]2O[C@H](CO)[C@@H](O)[C@@H](O)[C@H]2O)[C@H](F)[C@H](O)[C@@H]1O |
| 97 | 70905932 | O=P(O)(O)OC[C@@H]1O[C@H](O[C@H]2O[C@@H](CO)[C@@H](O)[C@@H](O)[C@H]2O)[C@H](F)[C@@H](O)[C@@H]1O |
| 98 | 70905914 | COP(=O)(OC)OC[C@H]1O[C@H](O[C@H]2O[C@H](CO)[C@@H](O)[C@H](O)[C@@H]2O)[C@@H](O)[C@H](O)[C@@H]1O |
| 99 | 70905911 | O=P(O)(O)OC[C@@H]1O[C@H](O[C@H]2O[C@@H](CO)[C@@H](O)[C@H](O)[C@H]2O)[C@H](O)[C@H](O)[C@@H]1O |
| 100 | 68982564 | O=P(O)(O)O[C@H]1[C@H](O)[C@@H](O)[C@H](OC[C@H](O)[C@H](O)[C@H](O)CO)O[C@@H]1CO |
| 101 | 68099456 | O=P(O)(O)OC[C@@H]1O[C@@H](O[C@@H]2O[C@@H](CO)[C@H](O)[C@@H](O)[C@@H]2O)[C@@H](O)[C@H](O)[C@H]1O |
| 102 | 67801750 | O=P(O)(O)O[C@@H]1O[C@H](CO[C@H]2O[C@H](CO)[C@H](O)[C@H](O)[C@H]2O)[C@@H](O)[C@H](O)[C@H]1O |
| 103 | 67716099 | O=P(O)(O)O[C@@H]([C@H](O[C@H]1O[C@H](CO)[C@@H](O)[C@H](O)[C@H]1O)[C@H](O)CO)[C@@H](O)CO |
| 104 | 66600013 | O=P(O)(O)O[C@H]1[C@H](O[C@H]2[C@H](O)[C@@H](O)[C@H](O)O[C@@H]2CO)O[C@H](CO)[C@H](O)[C@@H]1O |
| 105 | 57226062 | O=P(O)(O[C@@H]1O[C@H](CO)[C@@H](O)[C@H](O)[C@H]1O)O[C@@H]1O[C@H](CO)[C@@H](O)[C@H](O)[C@H]1O |
| 106 | 53881309 | O=P(O)(O)OC[C@H](O[C@@H]1O[C@H](CO)[C@@H](O)[C@H](O)[C@H]1O)[C@H](O)[C@H](O)CO |
| 107 | 53847998 | O=P(O)(O)O[C@H]1[C@@H](OC[C@H]2O[C@@H](O)[C@H](O)[C@@H](O)[C@@H]2O)O[C@H](CO)[C@H](O)[C@@H]1O |
| 108 | 53847997 | O=P(O)(O)O[C@H]1[C@H](OC[C@H]2O[C@@H](O)[C@H](O)[C@@H](O)[C@@H]2O)O[C@H](CO)[C@@H](O)[C@@H]1O |
| 109 | 49873682 | CO[P@@](=O)(O)OC[C@H]1O[C@H](O[C@H]2O[C@H](CO)[C@@H](O)[C@H](O)[C@H]2O)[C@H](O)[C@@H](O)[C@@H]1O |
| 110 | 49873670 | COP(=O)(OC)OC[C@H]1O[C@H](O[C@H]2O[C@H](CO)[C@@H](O)[C@H](O)[C@H]2O)[C@H](O)[C@@H](O)[C@@H]1O |
| 111 | 20831671 | O=P(O)(O)O[C@H]1[C@H](O)[C@@H](O)[C@H](O[C@H]2[C@H](O)[C@@H](O)[C@H](O)O[C@@H]2CO)O[C@@H]1CO |
| 112 | 11582571 | O=P(O)(O)O[C@@H]1O[C@H](CO)[C@@H](O[C@H]2O[C@H](CO)[C@@H](O)[C@H](O)[C@H]2O)[C@H](O)[C@H]1O |
| 113 | 11487007 | O=P(O)(O)OC[C@H]1O[C@H](OC[C@H]2O[C@@H](O)[C@H](O)[C@@H](O)[C@@H]2O)[C@H](O)[C@@H](O)[C@H]1O |
| 114 | 10949880 | O=P(O)(O)O[C@H]1[C@H](O)[C@@H](O)[C@@H](O[C@H]2O[C@H](CO)[C@@H](O)[C@H](O)[C@H]2O)O[C@@H]1CO |
| 115 | 440449 | O=P(O)(O)OC[C@H](O)CO[C@H]1O[C@H](CO)[C@H](O)[C@H](O)[C@H]1O |
| 116 | 442815 | O=P(O)(O)OC[C@H](CO)O[C@@H]1O[C@H](CO)[C@@H](O)[C@H](O)[C@H]1O |
| 117 | 10625889 | CO[C@@H]1O[C@H](CO)[C@H](O[C@H]2O[C@H](CO)[C@H](O)[C@H](O)[C@H]2O)[C@H](O)[C@H]1OCCOP(=O)(O)O |
| 118 | 10864814 | O=P(O)(O)O[P@](=O)(O)O[P@@](=O)(O)O[C@@H]1[C@@H](O)[C@@H](O[C@H]2O[C@H](CO)[C@@H](O)[C@H](O)[C@H]2O)O[C@H](CO)[C@H]1O |
| 119 | 10864815 | O=P(O)(O)O[P@](=O)(O)O[P@@](=O)(O)O[C@H]1[C@@H](O[C@H]2[C@H](O)[C@@H](O)[C@@H](O)O[C@@H]2CO)O[C@H](CO)[C@@H](O)[C@@H]1O |
| 120 | 10918396 | O=P(O)(O)O[C@H]1O[C@H](CO)[C@@H](O[C@@H]2O[C@H](CO)[C@H](O)[C@H](O)[C@H]2O)[C@H](O)[C@@H]1O |
| 121 | 10928408 | O=P(O)(O)O[14CH2][14C@H]1O[14C@H](O[14C@H]2[14C@@H](O)[14C@H](O)[14C@@H]([14CH2]O)O[14C@@H]2O)[14C@@H](O)[14C@@H](O)[14C@@H]1O |
| 122 | 10961632 | O=P(O)(O)OC[C@]1(O)OC[C@@H](O[C@@H]2O[C@H](CO)[C@@H](O)[C@H](O)[C@H]2O)[C@H](O)[C@H]1O |
| 123 | 11017326 | O=P(O)(O)O[P@](=O)(O)O[P@@](=O)(O)O[C@H]1[C@@H](O[C@H]2[C@H](O)[C@@H](O)[C@H](O)O[C@@H]2CO)O[C@H](CO)[C@@H](O)[C@@H]1O |
| 124 | 11050150 | O=P(O)(O)O[P@](=O)(O)O[P@@](=O)(O)O[C@@H]1O[C@H](CO)[C@@H](O[C@@H]2O[C@H](CO)[C@@H](O)[C@H](O)[C@H]2O)[C@H](O)[C@H]1O |
| 125 | 11060929 | O=P(O)(O)O[P@](=O)(O)O[P@@](=O)(O)O[C@@H]1O[C@H](CO)[C@@H](O[C@@H]2O[C@H](CO)[C@H](O)[C@H](O)[C@H]2O)[C@H](O)[C@H]1O |
| 126 | 11103841 | O=P(O)(O)O[P@](=O)(O)O[P@@](=O)(O)O[C@H]1O[C@H](CO)[C@@H](O[C@@H]2O[C@H](CO)[C@@H](O)[C@H](O)[C@H]2O)[C@H](O)[C@H]1O |
| 127 | 11177667 | O=P(O)(O)OC[C@H]1O[C@H](O[C@H]2O[C@@H](CO)[C@H](O)[C@@H](O)[C@@H]2O)[C@H](O)[C@@H](O)[C@@H]1O |
| 128 | 11498519 | O=P(O)(O)O[C@H]1O[C@@H](COCCOC[C@H]2O[C@@H](OCCO)[C@H](O)[C@@H](O)[C@H]2O)[C@@H](O)[C@@H](O)[C@@H]1O |
| 129 | 11549297 | O=P(O)(O)O[C@H]1O[C@@H](COCOC[C@H]2O[C@@H](OCCO)[C@H](O)[C@@H](O)[C@H]2O)[C@@H](O)[C@@H](O)[C@@H]1O |
| 130 | 17754190 | O=P(O)(O)OC[C@H]1O[C@H](O[C@@H]2O[C@H](CO)[C@@H](O)[C@H](O)[C@H]2O)[C@H](O)[C@@H](O)[C@@H]1O |
| 131 | 20831670 | O=P([O-])([O-])O[C@H]1[C@H](O)[C@@H](O)[C@H](O[C@H]2[C@H](O)[C@@H](O)[C@H](O)O[C@@H]2CO)O[C@@H]1CO |
| 132 | 23421895 | O=[P@]([O-])(O)O[C@H]1O[C@@H](COCCOC[C@H]2O[C@@H](OCCO)[C@H](O)[C@@H](O)[C@H]2O)[C@@H](O)[C@@H](O)[C@@H]1O |
| 133 | 25232871 | O=P(O)(O)O[C@H]1O[C@H](CO)[C@@H](O)[C@H](O)[C@@H]1O[C@@H]1O[C@H](CO)[C@@H](O)[C@H](O)[C@@H]1O |
| 134 | 25244273 | O=P([O-])([O-])OC[C@H]1O[C@@H](O[C@H]2[C@H](O)[C@@H](O)[C@H](O)O[C@@H]2CO)[C@H](O)[C@@H](O)[C@@H]1O |
| 135 | 44182624 | O=P(O)(O)O[C@@H]1O[C@H](CO)[C@@H](O[C@@H]2O[C@H](CO)[C@@H](OP(=O)(O)O)[C@H](O)[C@H]2O)[C@H](O)[C@H]1O |
| 136 | 44182626 | O=P(O)(O)O[C@H]1[C@H](O)[C@@H](O)[C@H](O[C@H]2[C@H](O)[C@@H](O)[C@@H](O)O[C@@H]2CO)O[C@@H]1CO |
| 137 | 44182628 | O=P(O)(O)O[C@@H]1O[C@H](CO)[C@@H](O[C@@H]2O[C@H](CO)[C@@H](O)[C@H](O)[C@H]2O)[C@H](O)[C@H]1O |
| 138 | 44606980 | CO[C@H]1O[C@H](CO)[C@@H](O)[C@H](O[P@@](=O)(O)O[C@H]2O[C@H](CO)[C@@H](O)[C@H](O)[C@@H]2O)[C@@H]1O |
| 139 | 72193696 | O=P(O)(O)OC[C@@H](CO)O[C@H]1O[C@H](CO)[C@@H](O)[C@H](O)[C@H]1O |
| 140 | 90657220 | O=P([O-])([O-])OC[C@H]1O[C@@H](O[C@H]2[C@H](O)[C@@H](O)[C@@H](O)O[C@@H]2CO)[C@H](O)[C@@H](O)[C@@H]1O |
| 141 | 90657221 | O=P(O)(O)OC[C@H]1O[C@@H](O[C@H]2[C@H](O)[C@@H](O)[C@@H](O)O[C@@H]2CO)[C@H](O)[C@@H](O)[C@@H]1O |
| 142 | 90657440 | O=P([O-])([O-])OC[C@H]1O[C@H](O[C@H]2[C@H](O)[C@H](O)CO[C@]2(O)CO)[C@H](O)[C@@H](O)[C@@H]1O |
| 143 | 90657441 | O=P(O)(O)OC[C@H]1O[C@H](O[C@H]2[C@H](O)[C@H](O)CO[C@]2(O)CO)[C@H](O)[C@@H](O)[C@@H]1O |
| 144 | 90657831 | O=P([O-])([O-])OC[C@H]1O[C@@H](O[C@H]2O[C@H](CO)[C@@H](O)[C@H](O)[C@H]2O)[C@H](O)[C@@H](O)[C@@H]1O |
| 145 | 90657832 | O=P(O)(O)OC[C@H]1O[C@@H](O[C@H]2O[C@H](CO)[C@@H](O)[C@H](O)[C@H]2O)[C@H](O)[C@@H](O)[C@@H]1O |
| 146 | 90658244 | O=P([O-])([O-])OC[C@H]1O[C@@H](O[C@H]2[C@H](O)[C@@H](O)[C@H](O)O[C@@H]2CO)[C@H](O)[C@@H](O)[C@H]1O |
| 147 | 90658245 | O=P(O)(O)OC[C@H]1O[C@@H](O[C@H]2[C@H](O)[C@@H](O)[C@H](O)O[C@@H]2CO)[C@H](O)[C@@H](O)[C@H]1O |
| 148 | 90658275 | O=P([O-])([O-])OC[C@H]1O[C@H](O[C@H]2[C@H](O)[C@@H](O)[C@H](O)O[C@@H]2CO)[C@H](O)[C@@H](O)[C@@H]1O |
| 149 | 90658276 | O=P(O)(O)OC[C@H]1O[C@H](O[C@H]2[C@H](O)[C@@H](O)[C@H](O)O[C@@H]2CO)[C@H](O)[C@@H](O)[C@@H]1O |
| 150 | 91872455 | O=P(O)(O)O[C@H]1O[C@H](CO)[C@@H](O[C@H]2O[C@H](CO)[C@@H](O)[C@H](O)[C@H]2O)[C@H](O)[C@H]1O |
| 151 | 97299411 | O=P(O)(O)O[C@@H]1O[C@H](CO)[C@@H](O[C@@H]2O[C@H](CO)[C@H](O)[C@H](O)[C@H]2O)[C@H](O)[C@H]1O |
| 152 | 101404976 | O=P(O)(O)OC[C@H]1O[C@H](O)[C@H](O)[C@@H](O)[C@@H]1O[C@H]1O[C@H](CO)[C@@H](O)[C@H](O)[C@H]1O |
| 153 | 101450524 | O=P(O)(O)OC[C@H]1O[C@H](O)[C@H](O)[C@@H](O)[C@@H]1O[C@@H]1O[C@H](CO)[C@@H](O)[C@H](O)[C@H]1O |
| 154 | 101715900 | O=P(O)(O)OC[C@H]1O[C@H](O[C@@H]2[C@H](O)CO[C@](O)(CO)[C@H]2O)[C@H](O)[C@@H](O)[C@@H]1O |
| 155 | 101715901 | O=P(O)(O)OC[C@H]1O[C@H](O[C@@H]([C@H](O)[C@@H](O)CO)[C@H](O)CO)[C@H](O)[C@@H](O)[C@@H]1O |
| 156 | 101715902 | O=P(O)(O)OC[C@H]1O[C@H](O[C@@H]2CO[C@](O)(CO)[C@@H](O)[C@@H]2O)[C@H](O)[C@@H](O)[C@@H]1O |
| 157 | 101715903 | O=P(O)(O)OC[C@H]1O[C@H](OC[C@@]2(O)OC[C@@H](O)[C@@H](O)[C@@H]2O)[C@H](O)[C@@H](O)[C@@H]1O |
| 158 | 101715905 | O=P(O)(O)OC[C@H]1O[C@H](OC[C@H]2O[C@@H](O)[C@H](O)[C@@H](O)[C@@H]2O)[C@H](O)[C@@H](O)[C@@H]1O |
| 159 | 102245203 | O=P(O)(O)O[C@H]1O[C@H](CO[C@H]2O[C@H](CO)[C@@H](O)[C@H](O)[C@@H]2O)[C@@H](O)[C@H](O)[C@@H]1O |
| 160 | 102398977 | O=P(O)(O)OC[C@H]1O[C@H](OC[C@H]2O[C@H](O)[C@H](O)[C@@H](O)[C@@H]2O)[C@H](O)[C@@H](O)[C@H]1O |
| 161 | 124932995 | O=P(O)(O)O[C@H]1O[C@@H](CO)[C@H](O[C@@H]2O[C@H](CO)[C@@H](O)[C@H](O)[C@@H]2O)[C@@H](O)[C@@H]1O |
| 162 | 124932996 | O=P(O)(O)O[C@H]1O[C@@H](CO)[C@H](O[C@@H]2O[C@H](CO)[C@H](O)[C@@H](O)[C@@H]2O)[C@@H](O)[C@@H]1O |
| 163 | 124932997 | O=P(O)(O)O[C@H]1O[C@@H](CO)[C@H](O[C@@H]2O[C@H](CO)[C@@H](O)[C@@H](O)[C@@H]2O)[C@@H](O)[C@@H]1O |
| 164 | 124932998 | O=P(O)(O)O[C@H]1O[C@@H](CO)[C@H](O[C@@H]2O[C@H](CO)[C@H](O)[C@H](O)[C@@H]2O)[C@@H](O)[C@@H]1O |
| 165 | 132934362 | O=P(O)(O)OC[C@H]1O[C@H](O[C@H]2O[C@H](CO)[C@@H](O)[C@H](O)[C@H]2O)[C@@H](O)[C@@H](O)[C@@H]1O |
| 166 | 148247164 | O=P(O)(O[C@H]1O[C@H](CO)[C@@H](O)[C@H](O)[C@@H]1O)O[C@H]1O[C@H](CO)[C@@H](O)[C@H](O)[C@@H]1O |

**Supp. Table S2**. N-(phenylthio) phthalimide (NPP) analogs.

| **N** | **CID PubChem** | **Smiles** |
| --- | --- | --- |
| 1 | 28777 | O=C1c2ccccc2C(=O)N1SC1CCCCC1 |
| 2 | 229606 | O=C1c2ccccc2C(=O)N1S(=O)(=O)c1ccccc1 |
| 3 | 97623 | O=C(c1ccccc1)N1C(=O)c2ccccc2C1=O |
| 4 | 214133 | Cc1ccc(S(=O)(=O)N2C(=O)c3ccccc3C2=O)cc1 |
| 5 | 118724034 | O=C(CCCCc1ccccc1)n1sc2ccccc2c1=O |
| 6 | 118724031 | O=C(CCCc1ccccc1)n1sc2ccccc2c1=O |
| 7 | 118724028 | O=C(CCc1ccc(F)cc1)n1sc2ccccc2c1=O |
| 8 | 118724027 | O=C(CCc1cccc(F)c1)n1sc2ccccc2c1=O |
| 9 | 118724026 | O=C(CCc1ccccc1F)n1sc2ccccc2c1=O |
| 10 | 118724022 | O=C(CCc1ccccc1)n1sc2ccccc2c1=O |
| 11 | 118724016 | O=C(Cc1ccc(F)cc1)n1sc2ccccc2c1=O |
| 12 | 118724015 | O=C(Cc1cccc(F)c1)n1sc2ccccc2c1=O |
| 13 | 118724014 | O=C(Cc1ccccc1F)n1sc2ccccc2c1=O |
| 14 | 118724008 | Cc1ccccc1CC(=O)n1sc2ccccc2c1=O |
| 15 | 118724007 | O=C(Cc1ccccc1)n1sc2ccccc2c1=O |
| 16 | 4913929 | CCCCSN1C(=O)c2ccccc2C1=O |
| 17 | 4116575 | O=C1c2ccccc2C(=O)N1SCc1ccccc1 |
| 18 | 301954 | CCSN1C(=O)c2ccccc2C1=O |
| 19 | 161114 | Cc1ccc(SN2C(=O)c3ccccc3C2=O)cc1 |
| 20 | 38348 | CCCSN1C(=O)c2ccccc2C1=O |
| 21 | 10976806 | O=C1c2ccccc2C(=O)N1SCl |
| 22 | 5127161 | O=C1c2ccccc2C(=O)N1Sc1ccccc1 |
| 23 | 4101460 | CSN1C(=O)c2ccccc2C1=O |
| 24 | 817973 | O=C1c2ccccc2C(=O)N1Sc1ccc(Cl)cc1 |
| 25 | 640880 | O=C(c1ccccc1)n1sc2ccccc2c1=O |
| 26 | 129694483 | O=C1c2cccc(C3CCCCC3)c2C(=O)N1S |
| 27 | 85090309 | Cc1ccccc1SN1C(=O)c2ccccc2C1=O |
| 28 | 85090307 | CCc1ccccc1SN1C(=O)c2ccccc2C1=O |
| 29 | 85090305 | Cc1cccc(C)c1SN1C(=O)c2ccccc2C1=O |
| 30 | 85090293 | CC(C)c1ccccc1SN1C(=O)c2ccccc2C1=O |
| 31 | 78201037 | O=C(C=Cc1ccccc1)n1sc2ccccc2c1=O |
| 32 | 71362308 | O=C1c2ccccc2C(=O)N1[S@](=O)Cc1ccccc1 |
| 33 | 21449896 | O=C(Cc1ccccc1)N1C(=O)c2ccccc2C1=O |
| 34 | 19710504 | O=C1c2ccccc2C(=O)N1S |
| 35 | 18673072 | CC(C)c1cccc(C(C)C)c1SN1C(=O)c2ccccc2C1=O |
| 36 | 12879125 | O=C1c2ccccc2C(=O)N1Sc1cccc2ccccc12 |
| 37 | 12761629 | CCCSSN1C(=O)c2ccccc2C1=O |
| 38 | 12416354 | O=C1c2ccccc2C(=O)N1SSCc1ccccc1 |
| 39 | 3678810 | CCCCCSN1C(=O)c2ccccc2C1=O |
| 40 | 848877 | CC(C)(C)SN1C(=O)c2ccccc2C1=O |
| 41 | 590073 | O=C1c2ccccc2C(=O)N1[S@](=O)c1ccccc1 |
| 42 | 401488 | O=C1c2ccccc2C(=O)N1C(=S)c1ccccc1 |
| 43 | 304026 | CS(C)(C)N1C(=O)c2ccccc2C1=O |
| 44 | 300122 | O=C1c2ccccc2C(=O)N1N[S@](=O)c1ccccc1 |
| 45 | 155344786 | N#CSN1C(=O)c2ccccc2C1=O |
| 46 | 154378505 | CC(C)C(C)(C)SSN1C(=O)c2ccccc2C1=O |
| 47 | 154344016 | O=C1c2ccccc2C(=O)N1SC1CCCC1 |
| 48 | 154132760 | O=C1c2ccccc2C(=O)N1SC1CCCCCCCCCCC1 |
| 49 | 154119268 | Cc1cccc(SN2C(=O)c3ccccc3C2=O)c1 |
| 50 | 152739078 | O=C1c2ccccc2C(=O)N1OSc1ccc(F)cc1 |
| 51 | 151482082 | O=C1c2ccccc2C(=O)N1OSCc1ccccc1 |
| 52 | 151087111 | O=C1c2ccccc2C(=O)N1S(=O)(=O)c1ccccc1F |
| 53 | 150969766 | Cc1ccc(S(=O)(=O)S(=O)(=O)N2C(=O)c3ccccc3C2=O)cc1 |
| 54 | 150137284 | [N-]=[N+]=NSc1ccc(CCN2C(=O)c3ccccc3C2=O)cc1 |
| 55 | 149042394 | Cc1ccc(SON2C(=O)c3ccccc3C2=O)cc1 |
| 56 | 148686466 | O=C1c2ccccc2C(=O)N1SI |
| 57 | 148308503 | C=C/C=C\SN1C(=O)c2ccccc2C1=O |
| 58 | 144576952 | CN(C)Sc1ccc(CN2C(=O)c3ccccc3C2=O)cc1 |
| 59 | 142616542 | O=C1c2ccccc2C(=O)N1Cc1ccc(SCl)cc1 |
| 60 | 142466875 | CC(C)(C)c1ccccc1SN1C(=O)c2ccccc2C1=O |
| 61 | 142466860 | Cc1ccc(C(C)(C)C)cc1SN1C(=O)c2ccccc2C1=O |
| 62 | 142466859 | O=C1c2ccccc2C(=O)N1Sc1ccccc1F |
| 63 | 142466852 | CC(C)(C)c1cccc(SN2C(=O)c3ccccc3C2=O)c1 |
| 64 | 139911799 | O=C1c2ccccc2C(=O)N1SC1(Cl)CCCCC1 |
| 65 | 123931298 | O=C1c2ccccc2CN1Sc1ccccc1 |
| 66 | 101314971 | CCCCCCCSN1C(=O)c2ccccc2C1=O |
| 67 | 88930003 | O=C1c2cccc(S)c2C(=O)N1SC1CCCCC1 |
| 68 | 88768077 | O=C1c2ccccc2C(=O)N1C1(S)CCCCC1 |
| 69 | 88722275 | C=CCSN1C(=O)c2ccccc2C1=O |
| 70 | 87775700 | NSc1cccc2c1C(=O)N(SC1CCCCC1)C2=O |
| 71 | 87754427 | O=C1c2ccccc2C(=O)N1SC1(C2CCCCC2)CCCCC1 |
| 72 | 85865134 | O=C(CCCc1ccccc1)N1C(=O)c2ccccc2C1=O |
| 73 | 85779180 | O=C1c2ccccc2C(=O)N1SSC1CCCCC1 |
| 74 | 85090311 | CCc1cccc(CC)c1SN1C(=O)c2ccccc2C1=O |
| 75 | 85090304 | CC(C)c1cc(SN2C(=O)c3ccccc3C2=O)cc(C(C)C)c1 |
| 76 | 85090295 | O=C1c2ccccc2C(=O)N1Sc1ccc(C(F)(F)F)cc1 |
| 77 | 71327781 | CC(=O)SSSN1C(=O)c2ccccc2C1=O |
| 78 | 71319532 | CC(C)(SN1C(=O)c2ccccc2C1=O)c1ccccc1 |
| 79 | 71319531 | C[C@H](SN1C(=O)c2ccccc2C1=O)c1ccccc1 |
| 80 | 71319530 | CCC(C)(C)SN1C(=O)c2ccccc2C1=O |
| 81 | 71319527 | C[C@@H]1CCCC[C@H]1SN1C(=O)c2ccccc2C1=O |
| 82 | 71319526 | CCC(CC)SN1C(=O)c2ccccc2C1=O |
| 83 | 71319524 | CC(C)[C@@H](C)SN1C(=O)c2ccccc2C1=O |
| 84 | 71319523 | CC(C)(C)CSN1C(=O)c2ccccc2C1=O |
| 85 | 71319522 | CC(C)CCSN1C(=O)c2ccccc2C1=O |
| 86 | 70037494 | CC(C)(C)[S@@](=O)N1C(=O)c2ccccc2C1=O |
| 87 | 66575468 | O=C1c2ccccc2C(=O)N1Sc1ccc(F)cc1 |
| 88 | 56834079 | O=C1c2ccccc2C(=O)N1Sc1ccc(Br)cc1 |
| 89 | 53815820 | O=C1NC(=O)c2c1cccc2-c1cccc(S)c1 |
| 90 | 23332330 | O=C1c2ccccc2C(=O)N1Sc1ccccc1Cl |
| 91 | 21688766 | O=C1c2ccccc2C(=O)N1SS |
| 92 | 21453674 | O=C1c2ccccc2C(=O)N1Cc1ccc(S)cc1 |
| 93 | 21292508 | CC(C)CSN1C(=O)c2ccccc2C1=O |
| 94 | 20482987 | CCCCCCSN1C(=O)c2ccccc2C1=O |
| 95 | 18731430 | O=C1c2ccccc2C(=O)N1SS(=O)(=O)c1ccccc1 |
| 96 | 14912179 | C/C(SN1C(=O)c2ccccc2C1=O)=C(\Cl)c1ccccc1 |
| 97 | 14788989 | C/C(Cl)=C(/C)SN1C(=O)c2ccccc2C1=O |
| 98 | 13725112 | CC[C@@H](C)SN1C(=O)c2ccccc2C1=O |
| 99 | 13617675 | CCCCSSN1C(=O)c2ccccc2C1=O |
| 100 | 13096784 | CC(C)(C)c1ccc(SN2C(=O)c3ccccc3C2=O)cc1 |
| 101 | 12893984 | Cc1ccccc1S(=O)(=O)N1C(=O)c2ccccc2C1=O |
| 102 | 12879124 | O=C1c2ccccc2C(=O)N1Sc1ccc2ccccc2c1 |
| 103 | 12879121 | O=C1c2ccccc2C(=O)N1Sc1ccccc1Br |
| 104 | 12861857 | O=C(/C=C/c1ccccc1)N1C(=O)c2ccccc2C1=O |
| 105 | 12761630 | CC(C)SN1C(=O)c2ccccc2C1=O |
| 106 | 12711573 | O=C1c2ccccc2C(=O)N1SC(=S)c1ccccc1 |
| 107 | 12462255 | O=C1c2ccccc2C(=O)N1[S@](=O)C1CCCCC1 |
| 108 | 12416740 | O=C1c2ccccc2C(=O)N1SSc1ccccc1 |
| 109 | 12416739 | Cc1ccc(SSN2C(=O)c3ccccc3C2=O)cc1 |
| 110 | 11807463 | CCCC/C=C/SN1C(=O)c2ccccc2C1=O |
| 111 | 10957847 | O=C1c2ccccc2C(=O)N1S/C=C/CCc1ccccc1 |
| 112 | 10935370 | O=C(CSN1C(=O)c2ccccc2C1=O)c1ccccc1 |
| 113 | 10469075 | CC[C@H](C)CSN1C(=O)c2ccccc2C1=O |
| 114 | 4148442 | O=C1c2ccccc2C(=O)N1S(=O)(=O)c1ccc(F)cc1 |
| 115 | 2322409 | Cc1ccccc1C(=O)N1C(=O)c2ccccc2S1(=O)=O |
| 116 | 902343 | O=C1c2ccccc2C(=O)N1OCSc1ccccc1 |
| 117 | 640882 | O=C(/C=C/c1ccccc1)n1sc2ccccc2c1=O |
| 118 | 912887 | Cc1ccc(C)c(SN2C(=O)c3ccccc3C2=O)c1 |
| 119 | 5251789 | CC(C)(C)SSN1C(=O)c2ccccc2C1=O |
| 120 | 10060430 | C=CC(=C)CSN1C(=O)c2ccccc2C1=O |
| 121 | 10313747 | Cc1ccc([S@](=O)SN2C(=O)c3ccccc3C2=O)cc1 |
| 122 | 10403533 | O=C1c2ccccc2C(=O)N1S[S@](=O)Cc1ccccc1 |
| 123 | 10999788 | O=C1c2ccccc2C(=O)N1SC1=CCCCC1 |
| 124 | 13617672 | O=C1CCCC[C@@H]1SN1C(=O)c2ccccc2C1=O |
| 125 | 14494070 | O=C1c2ccccc2CN1S(=O)(=O)c1ccccc1 |
| 126 | 14788994 | O=C1c2ccccc2C(=O)N1S/C(=C/Cl)c1ccccc1 |
| 127 | 14912180 | C/C(SN1C(=O)c2ccccc2C1=O)=C(/Cl)c1ccccc1 |
| 128 | 15517830 | O=C1c2ccccc2C(=O)N1[S@](=O)CCc1ccccc1 |
| 129 | 17768156 | O=C1c2ccccc2C(=O)N1[S-] |
| 130 | 20502352 | O=c1c2ccccc2c(=O)[n+]1=S |
| 131 | 21727678 | C[C@@H](SN1C(=O)c2ccccc2C1=O)C(=O)c1ccccc1 |
| 132 | 25181126 | [2H]c1c([2H])c([2H])c(SN2C(=O)c3ccccc3C2=O)c([2H])c1[2H] |
| 133 | 53469378 | O=C1c2ccccc2C(=O)N1CCc1ccc(S)cc1 |
| 134 | 54578132 | C=C[C@@H](SN1C(=O)c2ccccc2C1=O)[C@H](C)c1ccccc1 |
| 135 | 71470934 | O=C(SSCc1ccccc1)N1C(=O)c2ccccc2C1=O |
| 136 | 85646630 | O=C1c2ccccc2C(=O)N1SC(=CCl)c1ccccc1 |
| 137 | 85763673 | O=C(CCc1ccccc1)N1C(=O)c2ccccc2C1=O |
| 138 | 86166960 | Cc1cccc(S(=O)(=O)N2C(=O)c3ccccc3C2=O)c1 |
| 139 | 86167046 | O=C1c2ccccc2C(=O)N1S(=O)(=O)c1ccc(C(F)(F)F)cc1 |
| 140 | 90472525 | Cc1ccc([S@@](=O)N2C(=O)c3ccccc3C2=O)cc1 |
| 141 | 90472526 | CC(C)S[S@](=O)N1C(=O)c2ccccc2C1=O |
| 142 | 92162227 | O=C1c2ccccc2C(=O)N1N[S@@](=O)c1ccccc1 |
| 143 | 92225614 | O=C1c2ccccc2C(=O)N1N[S@](=O)c1ccccc1 |
| 144 | 101547804 | O=C1c2ccccc2C(=O)N1S(=O)(=O)c1ccc(I)cc1 |
| 145 | 101634236 | O=C1c2ccccc2C(=O)N1S/C(=C/Cl)Cc1ccccc1 |
| 146 | 101655825 | CCC[C@@H](C)SN1C(=O)c2ccccc2C1=O |
| 147 | 131115486 | Cc1ccccc1C(=O)N1C(=O)c2ccccc2C1=O |
| 148 | 134117797 | Cc1cc(C(C)(C)C)ccc1SN1C(=O)c2ccccc2C1=O |
| 149 | 144601888 | O=C(Cc1ccccc1)N1C(=O)c2ccccc2[S@@]1=O |
| 150 | 144904855 | O=C1c2ccccc2C(=O)N1Cc1ccc(SF)cc1 |
| 151 | 144904861 | O=C1c2ccccc2C(=O)N1Cc1ccc(SS)cc1 |
